# Circulating tumour cell-derived xenograft as a preclinical platform for metastatic breast cancer

**DOI:** 10.1038/s41416-026-03468-0

**Published:** 2026-05-18

**Authors:** Zuzana Kahounová, Markéta Hrušková, Stanislav Drápela, Ondřej Naar, Ráchel Víchová, Jiří Navrátil, Pavel Fabian, Filip Zavadil Kokáš, Aleš Hampl, Jan Bouchal, Karel Souček

**Affiliations:** 1 Department of Cytokinetics, Institute of Biophysics of the Czech Academy of Sciences, Brno, Czech Republic; 2 International Clinical Research Center, St. Anne’s University Hospital, Brno, Czech Republic; 3 Department of Experimental Biology, Faculty of Science, Masaryk University, Brno, Czech Republic; 4 Department of Comprehensive Cancer Care, Masaryk Memorial Cancer Institute, Brno, Czech Republic; 5 Department of Oncological Pathology, Masaryk Memorial Cancer Institute, Brno, Czech Republic; 6 Department of Bioinformatics, RECAMO, Masaryk Memorial Cancer Institute, Brno, Czech Republic; 7 Department of Histology and Embryology, Faculty of Medicine, Masaryk University, Brno, Czech Republic; 8 Department of Clinical and Molecular Pathology, Institute of Molecular and Translational Medicine, Faculty of Medicine and Dentistry, Palacky University and University Hospital, Olomouc, Czech Republic; 9 Present Address: Department of Tumor Microenvironment and Metastasis, H. Lee Moffitt Cancer Center & Research Institute, Tampa, FL USA

**Keywords:** Breast cancer, Cancer models

## Abstract

**Background:**

Circulating tumour cells (CTCs) are mediators of cancer dissemination and the formation of metastasis, which is the leading cause of cancer-related deaths. Experimental models derived from CTCs contribute to understanding the biology of CTCs, their role in dissemination, and the discovery of potential drugs targeting CTCs.

**Methods:**

A xenograft was derived from CTCs isolated from a patient diagnosed with metastatic invasive ductal carcinoma of the breast. The characterisation of the CTCs-derived xenograft (CDX) was conducted through in vivo experimental metastatic assays, RNA-Seq, spectral flow cytometry, and drug sensitivity tests.

**Results:**

The CTCs-enriched fraction formed a CDX within 6 months, and its metastatic potential was confirmed. CDX cells were propagated in vitro, where the enrichment of CD44^+^/CD24^−^ breast cancer stem cells was confirmed. An RNA-Seq-based comparison of CDX with the primary tumour from the same patient unravelled substantial changes in genes related to cell growth, metabolism, and extracellular signalling. CDX and in vitro cell culture showed sensitivity to carboplatin. A partial response was also observed for vandetanib, which was selected through in silico analysis of transcriptomic data.

**Conclusions:**

We present and characterise a novel model derived from CTCs for understanding the plasticity and behaviour of CTCs and advanced breast cancer.

**Figure:**
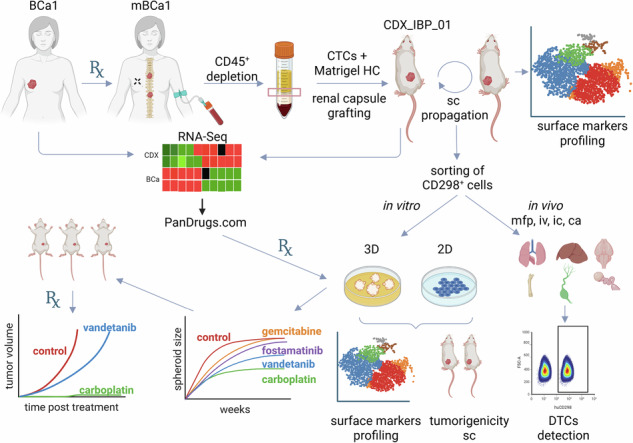
CDX_IBP_01 was established from the CTC-enriched fraction obtained from the patient with progressing breast cancer. Once stably re-transplanted and growing in vivo, the transcriptomes of CDX and archived primary BCa1 samples were compared. 2D and 3D in vitro cell cultures were established from sorted human cancer cells from an in vivo xenograft. Phenotypes of established models and their stability were characterised using spectral flow cytometry. The metastatic potential of CDX was evaluated in an in vivo assay. Finally, the applicability of the established model for in vivo and in vitro drug screening was evaluated. Created in https://BioRender.com.

## Background

Over the past two decades, analysis of liquid biopsy specimens, specifically circulating tumour cells (CTCs), circulating tumour DNA (ct-DNA), cell-free RNA, extracellular vesicles, and tumour-educated platelets, has become a promising tool for predicting the prognosis of metastatic patients, monitoring the progression of advanced cancer, or response to therapy [1]. Following advances in CTCs detection, counting, isolation, and characterisation techniques [2–4], the correlation of CTC mass with progression-free survival (PFS) and overall survival (OS) of patients with various malignancies serves as a prognostic marker for disease progression and a predictive marker of treatment response (reviewed in [5, 6]).

CTCs are released into the bloodstream from the primary tumour and metastatic sites. Due to the easy availability of CTCs from peripheral blood, CTCs have been widely studied as a valuable source of information on the metastatic cascade. The number of CTCs obtained from standard blood samples varies between tumour types and disease stages and is also influenced by the methods used for isolation and detection. The number is generally very low, in the order of units to the lower hundreds in a standard blood draw [7]. With the rapid development of specific technologies, these numbers are sufficient for single-cell-based analyses. Conversely, in instances where the phenotypic heterogeneity of a specific subpopulation of CTCs is to be analysed, or where the utilisation of CTCs is for drug screening, it becomes imperative to expand these cells. Preclinical models derived from CTCs, such as in vivo CTC-derived xenografts (CDXs) and in vitro cultures of CTCs, contribute to the understanding of the biology of CTCs, their role in dissemination, and the discovery of potential drugs targeting CTCs [8]. As CTCs are also derived from metastatic foci, CDXs derived from these foci fill a gap in relevant experimental models for studying metastatic disease. Although these models are promising, their derivation in vitro and in vivo remains challenging with numerous obstacles, as we discussed elsewhere [9].

Several studies describe the derivation of xenografts from CTCs of patients with breast cancer (BCa) using different approaches for both CTCs isolation/enrichment and in vivo expansion [10–12]. The various methods outlined in these publications emphasise the fact that there is presently no universal or standardised approach for the isolation and expansion of CTCs in vivo.

In the presented study, we delineate the derivation and characterisation of a xenograft from a CTC-enriched fraction isolated from the peripheral blood of a donor diagnosed with progressive multicentric invasive ductal carcinoma of the right breast. The generated CDX was transplantable in multiple passages in vivo and had metastatic capacity, as confirmed in an experimental metastatic assay. Human tumour cells were sorted from CDX and used to establish an in vitro cell culture. Cells, expanded either in monolayer or as 3D cell clusters in vitro, retained their tumorigenic capacity in vivo and were significantly enriched in CD44^+^/CD24^−^ BCa stem cells. Transcriptome comparison revealed substantial changes between the generated CDX and the corresponding patient’s primary tumour, which was obtained more than 8 years earlier. Finally, a small preclinical study was conducted to validate the relevance of both in vivo and in vitro models for drug testing, demonstrating the significant sensitivity of both models to carboplatin treatment. Overall, we present a unique xenograft derived from circulating tumour cells of a patient with progressive metastatic BCa that may serve as a useful model for further preclinical studies.

## Material and methods

### Patient information

**Fig. 1:**
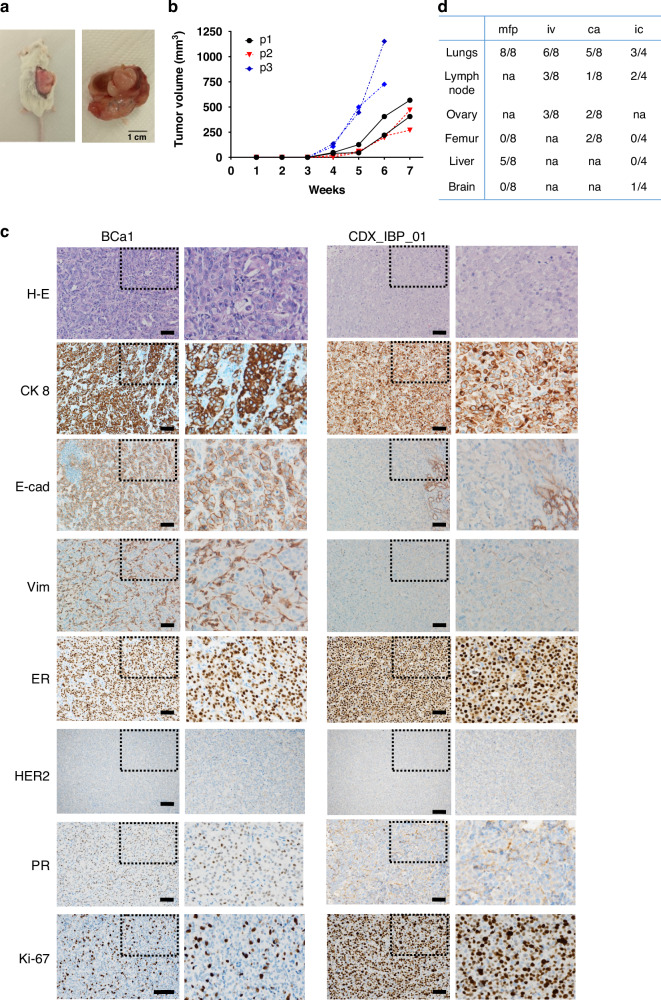
Established breast cancer CTCs-derived xenograft displays characteristics comparable to the primary tumour and is metastatic. **a** CDX_IBP_01 xenograft growing for 175 days after implantation of CTCs-enriched fraction in the subrenal capsule. Scale bar 1 cm. **b** Subcutaneous growth of established xenograft was monitored in three consecutive passages. Data from two representative mice per passage are presented. **c** Immunohistochemical comparison of established CDX and primary tumour from the same patient. H-E hematoxylin-eosin, CK 8 cytokeratin 8, E-cad E-cadherin, Vim vimentin, ER oestrogen receptor, HER2 human epidermal growth factor receptor 2, PR progesterone receptor. Scale bar 100 µm. Enlarged cut-outs are on the right side of the image of each particular marker. **d** Summary of results from 3 independent repetitions of the metastatic assay in vivo. *N* = 8 mice per mammary fat pad (mfp), tail vein (iv), and caudal artery (ca) injection, *n* = 4 per intracardiac (ic) injection. Results are presented as the number of animals with detectable human cancer cells/number of all animals injected. See Supplementary Fig. S2 for the full gating strategy and quantification of % of CD298^+^ cells in particular organs, and Supplementary Table S2 for detailed information about the number of cells analysed/detected.

Peripheral whole blood was obtained from a patient diagnosed with multicentric invasive ductal carcinoma of the right breast. The patient was diagnosed in 2011 at the age of 32 years (Supplementary Fig. S1A). The clinical characterisation of the primary tumour was as follows: Invasive carcinoma of no special type (NST) G2, pT3, pN1a (2 positive lymph nodes form 9), M0 (no distant metastases), L1 (lymphatic invasion) V0 (no venous invasion); ER 100%, PR 80%, Ki-67 59%, HER2 negative; see Fig. 1c for immunohistochemical analysis. Adjuvant chemotherapy with six cycles of TAC (docetaxel, doxorubicin, cyclophosphamide) was administered, followed by adjuvant radiotherapy of the chest wall and axilla, and adjuvant hormonal therapy with tamoxifen. In 2016, bone relapse was detected. Anastrozole (Arimidex) combined with ovariectomy (due to premenopausal status) was chosen as the first line of palliative treatment. After approximately 1 year of treatment, skeletal progression occurred again, and the therapy was changed to capecitabine chemotherapy. After 9 months of treatment, further skeletal progression was observed, followed by treatment with fulvestrant (Faslodex), vinorelbine (Navelbine), and gemcitabine (Gemzar). From September 2019, weekly paclitaxel was administered. Blood collection was performed in December 2019, 2 weeks after the last dose of paclitaxel, which had been discontinued due to skeletal progression. This was followed by another line of carboplatin treatment until March 2020, when it was terminated due to multiple relapses in the CNS. Palliative whole-brain radiotherapy was performed, but in May 2020, the patient died due to neurological progression. The tumour marker carcinoembryonic antigen (CEA) was always negative during treatment; only cancer antigen 15-3 (CA 15-3) was associated with disease progression. Initially, it decreased in response to the first two lines of palliative treatment, then it slowly and steadily increased. Both tumour markers were negative until relapse (Supplementary Fig. S1B).

### Blood processing, CTCs isolation, and in vivo implantation

Peripheral whole blood (7.5 ml) was harvested in a K_3_EDTA-containing tube. The CTCs-enriched fraction was obtained using incubation of the whole volume of blood with RosetteSep^TM^ Human CD45 Depletion Cocktail (#15162, STEMCELL^TM^ Technologies, Vancouver, Canada), followed by density gradient centrifugation with Lymphoprep^TM^ (#07801, STEMCELL^TM^ Technologies). The whole isolated CTC-enriched fraction was implanted in a 1:1 mixture with Matrigel® Basement Membrane Matrix High Concentration, LDEV-free (354248, Corning, NY, USA) in NOD.Cg-*Rag1*^*tm1Mom*^ *Il2rg*^*tm1Wjl*^/SzJ (NRG) female mouse (age 8.5 weeks, source The Jackson Laboratory, Bar Harbor, Maine, USA) in the right subrenal capsule. The number of CTCs in the enriched fraction was not evaluated before implantation. The mouse was regularly examined by manual palpation for signs of tumour growth. The growing tumour (CDX_IBP_01) was excised 175 days after implantation and re-transplanted subcutaneously in at least two NRG female mice, followed by several rounds of re-transplantation in vivo every 4–8 weeks.

### In vitro cell culture

To establish in vitro cell culture, CDX_IBP_01-derived single cell suspension deposited in liquid nitrogen was thawed and stained with an antibody detecting human CD298, a β3 subunit of the Na^+^/K^+^ ATPase used for discrimination of human cancer cells in xenograft models [13–16]. CD298^+^ cells were sorted and cultivated in vitro as 3D cell clusters embedded in Cultrex GFR BME Type II Path Clear (3533-010-02, R&D Systems, Minneapolis, USA) in cultivation medium described previously [17] with some modifications—Advanced DMEM (12491015, Thermo Fisher Scientific (TFS), Waltham, Massachusetts, USA) supplemented with 5% FBS (10270106, TFS), 10 mM HEPES, pH 7.4 (25245, Serva, Heidelberg, Germany), 1x Glutamax (35050-038, TFS), Gentamicin sulfate (50 µg/ml, 22185, Serva), 10 ng/ml hEGF (E9644, Merck, NJ, USA), 10 ng/ml FGF2 STAB (FGF2STAB000050, Enantis, Brno, Czech Republic), 1 µg/ml hydrocortisone (H0888, Merck), 10 µM Y-27632 dihydrochloride (sc-281642A, Santa Cruz Biotechnology, Texas, USA), 1mM N-acetyl-L-cysteine (A9165, Merck). When subculturing, a single-cell suspension was obtained by incubating Cultrex drops in 1 mg/ml Dispase (Dispase II, 04942078001, Merck) diluted in Advanced DMEM + 20% FBS for 45 min at 37 °C. After 20 min of incubation, Cultrex drops were manually disaggregated by pipetting several times up and down using a 1 ml tip. After another 25 min, the suspension was harvested, centrifuged at 300 × *g* for 5 min, and resuspended in TrypLE™ Select Enzyme (1X) without phenol red (12563029, TFS) for 2–3 min. It was then washed with Advanced DMEM + 20% FBS and spun at 300 × *g* for 5 min. The supernatant was resuspended in a cultivation medium and mixed with Cultrex to a final concentration of 75%. A 30–40 µl drop was placed on a culture dish (pre-heated at 37 °C) and the dish was immediately turned upside down and transferred to an incubator, where the drop was left to solidify for 15–20 min. After this time, the dish was turned back, and the complete cultivation medium was placed over the solid Cultrex drop. The cultivation medium was changed every 3–4 days. Depending on growth, 3D structures were subcultured every 2–4 weeks. Once growing as 3D spheroids, drops were disaggregated as described, and cells were seeded in adherent conditions in a monolayer in cultivation media described above. Fresh media was exchanged every 3–4 days. Cells were subcultured once reaching 70% confluency using incubation in EDTA/PBS for 4–5 min, followed by incubation with 0.5% Trypsin-EDTA (15400054, TFS) for 2 min.

Established cell culture and also xenograft were confirmed to be negative for specific Mycoplasma species, Squirrel Monkey Retrovirus, or Epstein-Barr virus by the Multiplex Cell Contamination Test (Multiplexion GmbH, Friedrichshafen, Germany). In vitro cell culture was also negative for contamination with cells of mouse origin.

### Spectral flow cytometry

Tumours were processed using mechanical and enzymatic digestion to prepare a single-cell suspension. Tumours were harvested in PBS and chopped into small fragments in a complete digestion medium containing DMEM (31966, TFS), 2 mg/ml collagenase type I (CLS1, LS004196, Worthington, Columbus, Ohio, USA), and 0.75 mg/ml Dispase II. Chopped fragments were mechanically disaggregated using a gentleMACS dissociator (Miltenyi Biotec, Bergisch Gladbach, Germany) with programmes m-imp-tumor-02 and m-imp-tumor-03, followed by incubation at 37 °C with gentle agitation. After 1 h, a gentleMACS dissociator was used again (programme m-imp-tumor-4), and DNase (10104159001, Merck) was added in a final concentration of 15 µg/ml. After 5 min of incubation at 37 °C, ice-cold PBS was added, and the suspension was centrifuged at 300 × *g* for 5 min at RT. The pellet was resuspended in 2 mL of EDTA/PBS and filtered through a CellTrics^TM^ 100 µm filter (04-0042-2318, Sysmex, Kobe, Japan). After centrifugation (300 × *g*, 5 min, RT), red blood cells were lysed using incubation with ammonium-chloride-potassium (ACK) lysis buffer for 5–10 min at 37 °C. After the final wash with PBS, up to 1 × 10^6^ cells were taken for flow cytometry analysis per sample. See Supplementary Table S1 for detailed information about antibodies and isotype controls used. Analysis was performed using an SP6800 spectral analyser (SONY Biotechnology, San Jose, USA). Cell doublets, aggregates, and debris were excluded from the analysis based on a dual-parameter dot plot [the pulse ratio (signal area/signal height; *y*-axis) versus signal area (*x*-axis) was displayed]. Dead cells were excluded from the analysis by staining with LIVE/DEAD^TM^ Fixable Yellow Dead Cell Stain (L34959, TFS). Data were analysed in FlowJo software (version 10.10.0, BD Bioscience, Franklin Lakes, New Jersey, USA).

### Spheroid formation assay

On day 0, CDX_IBP_01-derived 3D cell culture was harvested, and the single-cell suspension was prepared as described and manually diluted. 1, 10, 100, 5000, 1000, 2500, and 5000 cells per well in 60 µl of complete growth media were seeded in multiplicate in an ultra-low attachment 384-well plate (384-well Black/Clear Round Bottom Ultra-Low Attachment Spheroid Microplate, 4516, Corning). Images were taken every other day using ImageXpress® Micro High-Content Imaging System (Molecular Devices, San Jose, California, USA). Fresh medium (10–20 µl/well) was added weekly during spheroid cultivation for 21 days. Image analysis of individual spheroids was performed using MetaXpress software (v5.1, Molecular Devices). A pre-established and validated analysis pipeline was employed for image processing. Data points that did not correspond to the intended analysis criteria, such as the detection of multiple objects in the image, failure to identify the correct object or artifacts like fibres being included as part of the spheroid, were excluded from the results. Results were visualised using Prism 10.1.2 (GraphPad Software, L.L.C., Boston, MA, USA).

### In vivo tumorigenicity of cell culture

To examine the tumorigenicity of in vitro cell cultures, single-cell suspensions were prepared as described above. One hundred thousand cells per mouse were mixed 1:1 with Matrigel Basement Membrane Matrix High Concentration, LDEV-free (354248, Corning), and implanted subcutaneously in NRG female mice (age 10–11 weeks). Mice were monitored regularly, and tumour growth was measured weekly using a manual caliper. Tumour volume in mm^3^ was calculated as (length × width^2^) / 2, where the length is the larger dimension. Cells cultivated as 3D spheroids were in culture for 167 days (passage 7), and cells grown in 2D were in culture for 127 days (passage 6) when injected into animals for a tumorigenicity assay in vivo.

### In vivo metastatic assay

To conduct the metastatic assay, the single-cell suspension was prepared as described in Spectral flow cytometry section. The single cell suspension was stained for viability using LIVE/DEAD^TM^ Fixable Far Red Dead Cell Stain Kit (1:1000 in PBS, L10120, TFS), followed by washing with PBS and staining with CD298 FITC antibody and appropriate isotype control (see Supplementary Table S1 for details) for 20 min at 4 °C. After the final wash, viable/single cell/CD298^+^ cells were sorted using FACSAria II SORP system (BD Biosciences) equipped with five lasers (excitation wavelengths: 355, 405, 488, 561, and 639 nm). Sorted cells were washed with PBS and prepared for injections. The following injection sites were used: fourth mammary fat pad (500,000 cells in 100 µl in 1:1 PSB: Matrigel HC), tail vein (100,000 cells in 100 µl PBS), caudal artery (100,000 cells in 100 µl PBS), and intracardiac (100,000 cells in 50 µl PBS). Mice’s health status was regularly monitored, and the primary tumour in the mammary fat pad was measured weekly using a manual caliper.

For the detection of xenograft-derived cancer cells in circulation, mice were implanted in the mammary fat pad with fragments of xenograft tumour (approx. 3 × 3 × 3 mm) and allowed to grow for 9 weeks. At the endpoint, the primary tumour and lungs were collected and processed into single-cell suspensions as described below. Blood was collected from a deeply anaesthetised animal through cardiac puncture with a heparinized syringe. A total of 200 µl of whole blood was stained for viability and for surface CD298. Alternatively, approx. 1 ml of whole blood was taken from the animal with a heparinized syringe, diluted 1:1 with PBS, and centrifuged at 400 × *g* for 15 min with no brake. The intermediate layer ( = buffy coat) was harvested and processed similarly to whole blood. Red blood cell lysis was performed after the staining.

When harvested, a single-cell suspension was prepared from primary tumours and selected organs as described in Spectral flow cytometry section. Single-cell suspension from the liver and lungs was prepared similarly to the primary tumour, using different dissociation programmes on the gentleMACS dissociator (m_liver, or m-lungs_01, respectively). The single-cell suspension from the femur was prepared by flushing the cells from the femur using a 25 G needle and PBS. Single-cell suspension from the brain was prepared as follows [18]. Excised brain (one half) was placed in dissociation medium RPMI 1640 (72400054, TFS) supplemented with 2% FBS, 5 µg/ml insulin (I9278, Merck), 50 ng/ml gentamicin sulfate (22185, Serva), 0,2 mg/ml collagenase type 1, DNase (7 µg/ml), mechanically chopped, dissociated with gentleMACS dissociator (m_imp_brain_01) and incubated for 45 min at 37 °C on rotator. Then, the suspension was washed with ice-cold PBS, centrifuged (300 × *g*, 5 min), and incubated with 0.0025% trypsin supplemented with DNase (15 µg/ml). After centrifugation with a dissociation medium, the suspension was dissociated in PBS and filtered through a 100 µm filter (CellTrics^TM^, 100 µm, Blue, Sysmex). Myelin was removed by gradient centrifugation using 20%/80% Percoll (17-0891-02, Cytiva, Marlborough, Massachusetts) in PBS (1200 × *g*, 30 min, 4 °C, with no brakes during deceleration). Collected cells were washed in ice-cold PBS (300 × *g*, 5 min), and red blood cells were lysed with ACK lysis buffer (5 min, 37 °C).

The single cell suspension was stained with viability dye LIVE/DEAD^TM^ Fixable Yellow Dead Cell Stain Kit (L34959, TFS) or LIVE/DEAD^TM^ Fixable Aqua Dead Cell Stain Kit (L34966, TFS), followed by a cocktail of primary antibodies and appropriate isotype controls (see Supplementary Table 1)—mouse lineage markers CD45, CD31, Ter-119, and human-specific markers CD298. CDX-derived cancer cells were identified as viable/single cells/without debris/mouse lineage markers negative/human CD298 positive. Analysis was performed on an Attune^TM^ Acoustic Focusing Cytometer equipped with 2 lasers (405 nm, 488 nm) (1^st^ generation, TFS), and data were visualised and analysed using FlowJo™ Software.

### In vivo drug study

NRG female mice (age 7–9 weeks) were implanted with CDX_IBP_01 tumour fragments into the mammary fat pad. Seven weeks after implantation, when all tumours were detectable and growing, the mice were randomly divided into groups so that the mean tumour size was comparable in each group, and treatment began. Animals with tumours larger than 500 mm³ or animals without tumours were excluded from the experiment before the start of the treatment. Carboplatin (50 mg/kg, HY-17393, MedChem Express, New Jersey, USA) [19] was diluted in 0.9% NaCl and administered intraperitoneally (ip) once per week for 3 weeks. Vandetanib (25 mg/kg, HY-10260, MedChem Express) [20] was diluted in 50% PEG 300 (HY-Y0873, MedChem Express) + 50% 0.9% NaCl and administered via oral gavage (og) daily for 3 weeks. Control mice received 50% PEG 300 + 50% 0.9% NaCl og. Mice were regularly weighed; tumour volume was measured twice a week using a manual caliper. The experiment was terminated after 3 weeks, or earlier if the primary tumour size reached approximately 2 cm³ or if the health status of the mice deteriorated. The experiment was performed in two independent repetitions, with *n* = 3 mice per group in the first repetition and *n* = 5 mice in the second repetition. Note that some mice had to be terminated earlier, before the endpoint of 21 days of treatment. Only mice that had been on treatment for at least 2 weeks were included in the analysis: *n* = 7 for vehicle treatment, *n* = 8 for carboplatin treatment, and *n* = 7 for vandetanib treatment. At the endpoint, lungs were harvested, processed for single cell suspension as described in In vivo metastatic assay section, and analysed for the presence of disseminated cancer cells using flow cytometry.

### In vitro drug study

CDX_IBP_01 cell culture was dissociated into a single cell suspension, and 2500 cells were seeded per well of a 384-well ultra-low attachment plate (Corning® 384-well Black/Clear Round Bottom Ultra-Low Attachment Spheroid Microplate, with Lid, Sterile, #4516). After 7 days of cultivation, when 3D clusters formed, the selected treatment was applied. The treatment was repeated after another 7 days of cultivation. The experiment was ended 21 days after initial cell seeding. Spheroids were regularly monitored using ImageXpress® Micro High-Content Imaging System (Molecular Devices). Spheroid area was measured and data for treated groups were normalised to data obtained for control groups. Data were processed as described in Spheroid formation assay section. Control groups were treated with appropriate vehicles—water for gemcitabine and carboplatin and with DMSO in concentrations of 0.1% and 0.125% for fostamatinib and vandetanib, respectively. Gemcitabine hydrochloride (ND09343, Carbosynth, San Diego, California, USA), and carboplatin (HY-17393, MedChem Express, New Jersey, USA) [19] were diluted in water, fostamatnib (HY-13038A, MedChem Express) and vandetanib were diluted in DMSO (HY-10260, MedChem Express). Viability of spheroids at the endpoint was analysed using CellTitre-Glo® Luminiscent Cell Viability Assay (G7572, Promega, Madison, Wisconsin, USA) and measured on Infinite 200 PRO (TECAN, Männedorf, Switzerland).

### RNA-Seq analysis and data analysis

For RNA-Seq analysis, a single cell suspension was prepared from subcutaneously growing CDX. Human EpCAM-positive cells were isolated from a single cell suspension prepared from CDX_IBP_01 as described previously using CD326 (EpCAM) MicroBeads, human (130-061-101, Miltenyi Biotec). RNA from the EpCAM-enriched fraction from CDX_IBP_01 and frozen primary tumour sample (BCa1) from the same patient was isolated using the RNeasy Micro Plus Kit (74034, Qiagen, Hilden, Germany). A deep-frozen sample of the patient´s primary tumour was obtained from the BBMRI.cz, the Network of Czech Biobanks and processed similarly to the xenograft sample. Samples and data processing were done as we described elsewhere [21]. RNA sequencing data obtained from two biological replicates per group were aligned to the hg38 reference genome [22] using the HISAT2 aligner [23]. Reads mapped to gene regions were subsequently quantified with FeatureCounts [24], and differential expression analysis was performed using DESeq2 [25]. Genes were considered differentially expressed if they had an adjusted *p* value (*p*adj) <0.05 and a fold change >2.

Protein-coding genes were filtered and subjected to RNA-seq data analysis using published and well-described InteractiVenn [26], VolcaNoseR [27] and iDEP [28] integrated web online tools. Gene set enrichment analysis (GSEA) plot was generated using GSEA software (MSigDB 2024.1) and collection of Hallmarks, KEGG, and MitoCarta3.0 gene sets databases. MitoCarta3.0 gene set, established from mass spectrometry of mitochondria isolated from fourteen tissues, assessed protein localisation through large-scale GFP tagging/microscopy, and integrated these results with six other genome-scale datasets of mitochondrial localisation, using a Bayesian approach, was downloaded from Broad Institute [29]. Dot plots were derived from gene sets from Gene Ontology. After the analysis, pathways were filtered based on an FDR cutoff (0.05). Then the significant pathways were sorted by FDR. Heatmaps were generated using *z*-score and Morpheus (Broad Institute) online tool. The DEG genome location was generated using iDEP and scanning the genome with sliding windows. For all genes in a window/region, whether the mean of FC of these genes is zero was tested using a *t*-test. All genes analysed by DESeq2 were included in this analysis.

### Immunohistochemical analysis

Formalin-fixed paraffin-embedded (FFPE) tissue specimens were prepared according to standard histopathological procedures. Immunohistochemical detection of selected protein markers was performed using the Ventana Benchmark Ultra Plus (ROCHE) or PT Link (Agilent Dako), with an indirect immunohistochemical method. Antibodies and conditions used for staining are listed in Supplementary Table S5.

### Statistical analysis

Statistical analysis was performed with Prism 10.1.2 (GraphPad Software, L.L.C.).

### The use of generative AI and AI-assisted technologies

Grammarly (v1.2.173.1702), DeepL Translator, and DeepL Write, available online, were used for linguistic and stylistic proofreading.

### Ethics approval and informed consent

The study, including blood collection from patients with metastatic breast cancer, was approved by the Ethical Committee of the Masaryk Memorial Cancer Institute (MOU 319 690). The experiments were conducted with the understanding and informed consent of the patients. All animal experiments were conducted with the approval of the Academy of Sciences of the Czech Republic (AVCR 2459/2021 SOVII, AVCR 615/2024 SOVII, AVCR 7282/2024 SOVII), overseen by the ethical committee of the Institute of Biophysics of the Czech Academy of Sciences, performed by certified individuals (ZK, RV, MH, SD), and carried out following relevant guidelines and regulations.

## Results

### Derivation of xenograft from circulating tumour cells of a patient with metastatic BCa

The whole peripheral blood was obtained from a patient diagnosed with multicentric invasive ductal carcinoma of the right breast, approximately 8.5 years after the initial diagnosis and following surgical resection of the primary tumour. At the time of blood collection, the disease had already advanced, and the patient had undergone multiple cycles of chemotherapy (Supplementary Fig. S1A). The tumour marker CEA was always negative during treatment; only CA 15-3 was associated with disease progression. Initially, it decreased in response to the first two lines of palliative treatment, then it slowly and steadily increased. Both tumour markers were negative until relapse (Supplementary Fig. S1B). The patient succumbed to death 6 months after the blood was collected.

The CTCs-enriched fraction was isolated from the peripheral whole blood on the day of harvest as described in the “Materials and methods” section. The entire fraction was immediately implanted in a subrenal capsule [30] in an immunodeficient NRG female mouse without evaluating the number of CTCs in the fraction before implantation. The tumour growing in the renal capsule was excised 175 days after implantation (Fig. 1a and Supplementary Fig. S1C) and then serially retransplanted subcutaneously in other NRG female mice, with a comparable growth rate (Fig. 1b). The immunohistochemical analysis revealed comparable characteristics between the established CDX_IBP_01 (hereafter referred to as CDX) and the primary tumour, specifically positivity for the cancer cell marker cytokeratin 8 (CK8), oestrogen receptor (ER), and negativity for human epidermal growth factor receptor 2 (HER2) (Fig. 1c). In contrast, E-cadherin expression was partially lost, and vimentin expressions was completely lost in CDX compared to the primary tumour. Moreover, while the fraction of cancer cells from the primary tumour was positive for progesterone receptor (PR, 80%), the established xenograft was completely negative for PR. Xenograft also displays increased positivity for Ki-67, indicating its high proliferative capacity. Together, we successfully established CDX with confirmed human origin and preservation of some characteristics of the primary tumour.

### The CTC-derived xenograft possesses metastatic capacity

Given the phenotypic differences observed between the CDX and the primary tumour, we hypothesised that these changes may result from adaptations tumour cells undergo during metastatic progression. To investigate this, we conducted an in vivo experimental metastasis assay to assess the metastatic potential of the CDX model. CD298^+^ human cancer cells were sorted from dissociated CDX and injected into female NRG mice via four different routes, including orthotopic mammary fat pad (mfp), intravenously in the tail vein (iv), in a caudal artery (ca), and intracardially (ic). 8 to 13 weeks after cancer cells were injected, selected organs, anticipated to be infiltrated based on the route of injection, were harvested. In parallel, control organs from the intact animal were harvested and analysed to set the correct gating strategy for flow cytometry (Supplementary Fig. S2A, B). Upon orthotopic injection, the human cancer cells were present at high numbers in the lungs and, to a lesser extent, in the livers (Fig. 1d, Supplementary Fig. S2C and Supplementary Table S2). No metastatic cells were detected in the femur or brain. Following the caudal artery injection, metastases were present in the femur, lungs, and lymph nodes. After intravenous injection, infiltrated human cancer cells were detected in the lungs and lymph nodes. Lastly, the left cardiac ventricle injection resulted in metastatic dissemination in the brain of one animal.

To further prove the metastatic capacity of our model, we detected xenograft-derived cancer cells also in the blood of mice harbouring a primary tumour. Fragments of xenograft were implanted in the mammary fat pad, and paired samples of primary tumour, blood, and lungs from each animal were inspected for the presence of human cancer cells by detection of human cell-specific surface marker CD298. As shown in Supplementary Fig. S2D, CD298^+^ human cancer cells were detected in primary tumour, blood, and lungs. Further, we detected expression of surface molecule Trop-2, a marker of cancer cell plasticity, as we described previously [31]. Both flow cytometry (Supplementary Fig. S2E) and immunohistochemical analysis (Supplementary Fig. S2F) confirmed strong expression of Trop-2 in the primary tumour and Trop-2^+^ disseminated cancer cells in the lungs. In blood, partial positivity of CDX-derived circulating cancer cells for Trop-2 was detected. In summary, these results demonstrated that the xenograft established from CTCs of a patient with mBCa has metastatic capacity.

### CDX-derived in vitro cell cultures grow in both 2D and 3D conditions and exhibit tumorigenic properties

**Fig. 2:**
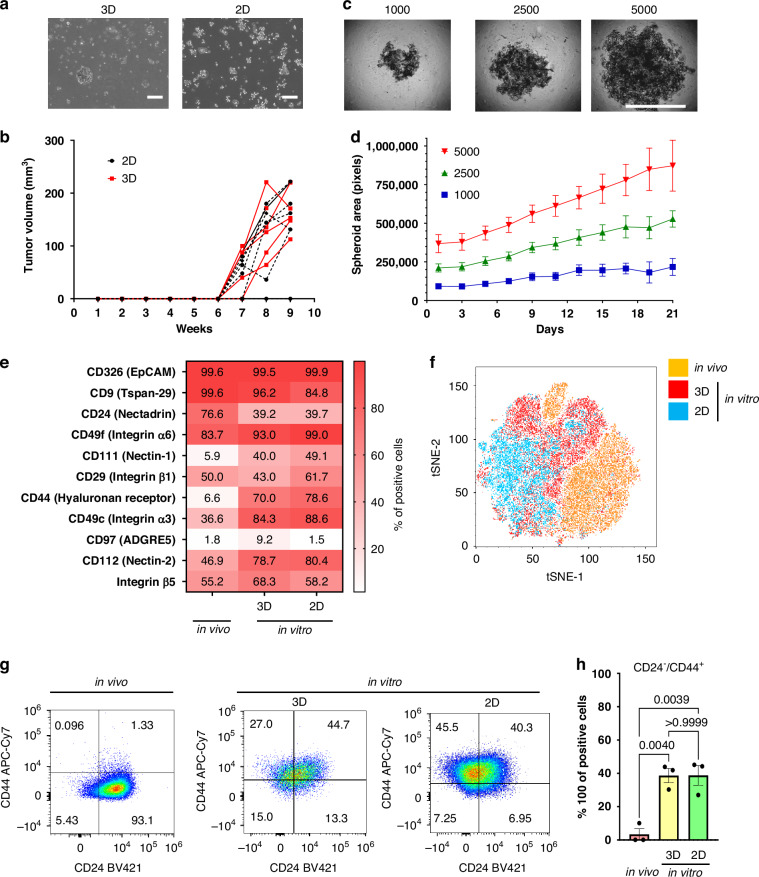
CDX-derived in vitro cell cultures are growing in both 2D and 3D conditions and are tumorigenic. **a** Morphology of in vitro cultures established as spheroids (3D) and monolayer cell cultures (2D). Scale bar 200 μm. **b** In vivo tumorigenicity assay of established in vitro cultures. Cell culture grown as spheroids (3D), or monolayer (2D) was injected subcutaneously in NRG female mice, and tumours were measured weekly using a manual caliper. **c** Spheroid formation assay using in vitro 3D cell culture. Representative images from seeded 1000, 2500, and 5000 cells per well are presented. Scale bar 300 µm. **d** Quantification of spheroids’ growth from **c** presented as an average spheroid area in pixels. *N* = 22 wells per cell density. **e** Analysis of selected surface epithelial-like (EpCAM, CD9, CD24, CD49f, CD111), and mesenchymal-like (CD29, CD44, CD49c, CD97, CD113, integrin β5) markers using spectral flow cytometry. Results are presented as the mean percentage of cells positive for each marker from three independent repetitions. See Supplementary Fig. S3 for statistical analysis. **f** t-SNE plot showing pooled in vivo xenograft, and in vitro 2D and 3D cell culture from one representative repetition. See Supplementary Fig. S4 for additional clustering analyses. **g** Detection of breast cancer stem cell subpopulation CD44^+^/CD24^−^ in in vivo xenograft and in vitro cell cultures. **h** Quantification of CD44^+^/CD24^−^ breast cancer stem cells. Results are presented as mean ± SEM from 3 independent repetitions. Statistical analysis was performed using ordinary One-way ANOVA and Tukey´s multiple comparison test.

To further expand our model, an in vitro cell culture was established from CDX. To this end, human CD298^+^ cells were sorted from the dissociated CDX and subsequently seeded in vitro in 3D conditions (Fig. 2a, left). Monolayer cell culture was also established after dissociation of growing spheroids from the extracellular matrix (Fig. 2a, right). Both established in vitro cell culture models were slow-growing, with subculturing performed every 2 to 4 weeks, and had been grown in culture for at least 3 months. To confirm that derived cell cultures still retain their tumorigenic potential, cell suspension dissociated from spheroids or monolayers was subcutaneously injected into immunodeficient female mice and monitored for tumour growth. Both cell cultures displayed maintained tumorigenic capacity with comparable growth rates (Fig. 2b); however, the rate was slower compared to the original CDX (Fig. 1b).

To provide further characterisation of the spheroid cell culture, a spheroid formation assay was conducted in non-adherent conditions. The objective of this experiment was to determine whether the formed spheroids originate from a single cell or result from the clustering of multiple cells, followed by their growth. 1, 10, 100, 500, 1000, 2500, and 5000 cells were seeded in each well in multiplicate and monitored every other day. Notably, robust 3D cell clusters formed only at seeding densities of 1000 cells per well or higher, while wells with fewer cells did not produce growing spheroids (Fig. 2c, d). In summary, we established CDX-derived cell cultures in both 2D and 3D setups that retained their tumorigenic potential in vivo and exhibited robust spheroid formation, which was dependent on a critical cell seeding density.

### Adaptation to in vitro conditions is associated with the enrichment of the CD44^+^/CD24^−^ cancer stem cell subpopulation

Adaptation of in vivo xenografts to in vitro conditions is often accompanied by phenotypic changes [32]. To describe the phenotype of the original xenograft and derived in vitro cell cultures, the expression of selected surface markers associated with epithelial-like (EpCAM/CD326, CD9/Tspan-29, CD24/Nectadrin, CD49f/Integrin α6, CD111/Nectin-1) and mesenchymal-like (CD29/Integrin β1, CD44/Hyaluronan receptor, CD49c/Integrin α3, CD97/ADGRE5, CD112/Nectin-2, and integrin β5) phenotypes was investigated using spectral flow cytometry. This panel was previously established in our laboratory and confirmed to characterise the EMT phenotype of BCa cells [31]. A surface marker CD298, specific for human cells, was added to the panel, and the aforementioned markers were analysed only on the CD298^+^ subpopulation in the case of a sample derived from in vivo conditions. Human cancer cells from in vivo conditions and derived in vitro cell cultures depicted a rare epithelial origin, with uniform positivity for epithelial markers (EpCAM, CD9, CD49f). In contrast, the expression of mesenchymal markers was variable, with generally higher positivity observed in in vitro cultures compared to in vivo conditions (Fig. 2e and Supplementary Figs. S3, S4A). Unsupervised FlowSOM analysis identified eight cellular populations that were present across 2D, 3D, and xenograft conditions (Supplementary Fig. 4B–D). Visualisation by t-SNE and FlowSOM topology demonstrated shared phenotypic structure among all samples. Quantitative analysis revealed condition-dependent shifts in the relative abundance of several populations, most notably Pop0 and Pop1, while the dominant population (Pop2) was maintained across all conditions (Supplementary Fig. S4E). No population was uniquely present or absent in any condition, indicating conservation of the overall cellular landscape with quantitative redistribution. Collectively, the samples share the same FlowSOM-defined populations, with differences reflecting shifts in relative abundance rather than the emergence of unique populations. Patient-derived xenografts have previously been shown to facilitate more accurate identification of cancer stem cells (CSCs) and tumour hierarchies, which is essential for understanding tumour biology and developing effective, personalised therapies [32]. Therefore, we investigated the presence of a stem cell population with the CD44^+^/CD24^−^ phenotype in our models. Interestingly, a sample derived from in vivo conditions, characterised by uniform positivity for CD24 and negativity for CD44 surface antigens, almost entirely lacks cells with the phenotype of BCa stem cells (Fig. 2g, in vivo). On the contrary, cell cultures from both in vitro conditions showed enrichment in cells with CSC phenotype (Fig. 2g, h). Together, these results indicate that adaptation to in vitro conditions is associated with increased expression of mesenchymal markers and a significant increase in CD44^+^/CD24^−^ CSC subpopulation.

### Transcriptomic comparison between matched primary tumour and CTC-derived xenograft

**Fig. 3:**
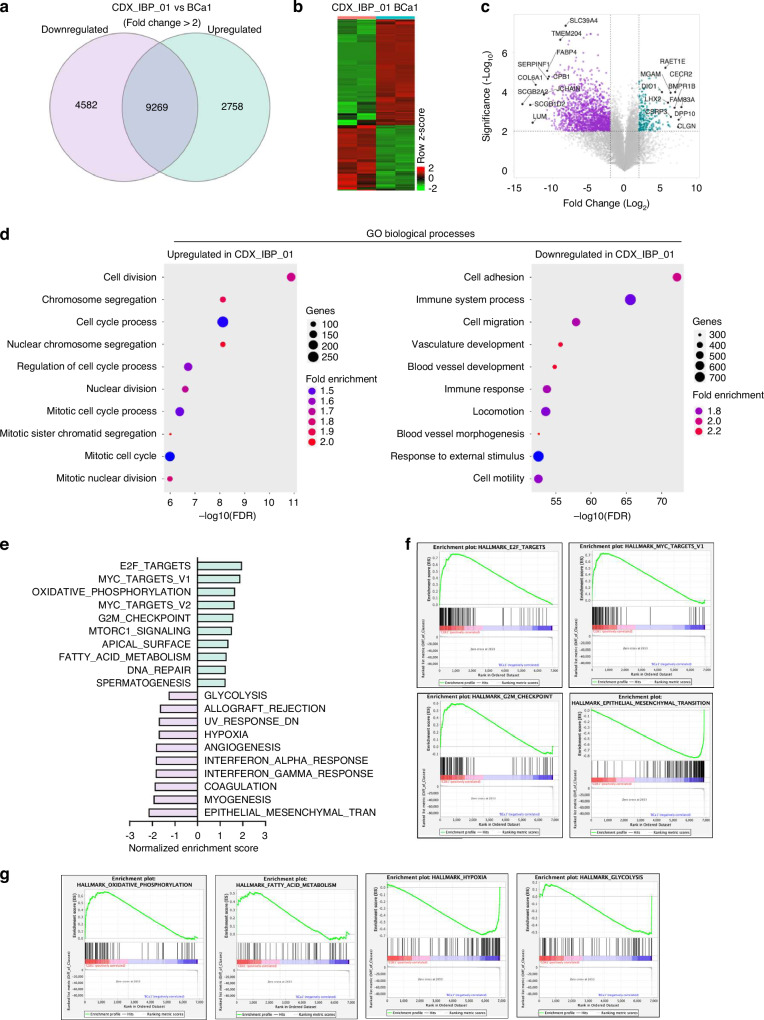
Transcriptomic analysis reveals a substantial disparity between CDX_IBP_01 and primary tumour gene expression profiles. **a** Venn diagram portraying genes downregulated and upregulated in CTCs-derived xenograft (CDX_IBP_01) relative to the primary tumour (BCa1) (Fold Change >2) determined by RNA-seq. **b** Heatmap representing *z*-score for top 5000 genes deregulated in CDX_IBP_01 vs. BCa1. **c** Volcano plot depicting the distribution of differentially expressed genes with the 10 most downregulated and 10 most upregulated genes within CDX_IBP_01 relative to BCa1. **d** Dot plot representing Gene Ontology Biological Processes enriched (left) or lost (right) within CDX_IBP_01 relative to BCa1. The dot size represents the number of genes of a particular biological process; the colour intensity represents the fold enrichment (CDX_IBP_01 vs. BCa1), and the *x*-axis determines the −log10 false discovery rate. **e** List of the 10 most positively- and negatively enriched gene sets from the Hallmarks database. **f** GSEA plots depicting the most deregulated Hallmarks biological processes between CDX_IBP_01 and BCa1 related to cellular signalling. **g** GSEA plots depicting the most deregulated Hallmarks biological processes between CDX_IBP_01 and BCa1 related to metabolism.

To date, only several studies have conducted transcriptomic comparisons between matched BCa primary tumours and CDXs. In this study, we employed RNA-seq technology to characterise transcriptomic changes between a primary tumour sample and paired CDX from the same patient. Initial analysis revealed extensive gene deregulation between these two matched models. Remarkably, over 44% of all protein-coding genes were differentially expressed, with approximately 17% upregulated and 27% downregulated in xenograft compared to the primary tumour, using a fold change threshold of >2 (Fig. 3a, b). Further analysis revealed the most differentially expressed genes between xenograft and primary tumour (Fig. 3c). Gene ontology analysis showed that genes upregulated in CDX relative to primary tumour (BCa1) are associated with biological processes related to cell division, proliferation, and cell cycle progression. Conversely, genes downregulated in CDX were predominantly linked to cell adhesion, migration, and immune response (Fig. 3d and Supplementary Fig. S5A). These molecular changes were consistently positively or negatively enriched in CDXs, as demonstrated by Gene Set Enrichment Analysis (GSEA) using Hallmark gene sets (Fig. 3e, f). In addition to these biological processes, Hallmark GSEA analysis uncovered a potential metabolic shift between CDX and primary tumour. While the primary tumour appeared to favour glycolysis associated with hypoxia, CDX exhibited a preference for fatty acid metabolism followed by oxidative phosphorylation (OXPHOS), suggesting increased mitochondrial demand (Fig. 3d, g). This phenomenon was corroborated by further analysis showing that upregulated genes in CDX were enriched in molecular processes such as OXPHOS and electron transport chain, as well as cellular components including the mitochondrial inner membrane (Supplementary Fig. S5B, C). Moreover, genes associated with mitochondrial function, mass, and fitness were significantly upregulated in CDX compared to the primary tumour (Supplementary Fig. S6A–D). Lastly, KEGG GSEA analysis indicated that genes downregulated in xenograft were associated with extracellular matrix interaction and focal adhesion (Supplementary Fig. S6E). This finding suggests a loss of epithelial cell features, consistent with the phenotypic changes accompanying circulating tumour cells. In conclusion, our results demonstrate that the transcriptome of CDX_IBP_01 differs substantially from that of the primary tumour BCa1. These differences encompass changes in cell growth, metabolism, and extracellular signalling, providing valuable insights into the molecular adaptations of circulating tumour cells. Next, the significant number of therapy cycles, both hormonal and chemotherapy, might also have an impact on the observed differences between the primary tumour and the xenograft.

### The established CDX is a versatile platform for preclinical evaluation of cancer treatments in vivo and in vitro

To showcase the utility of our CDX model for drug testing, we designed a small preclinical study. We took advantage of the publicly available bioinformatic platform PanDrugs (www.pandrugs.com, version 2024.06) [33, 34] and used it to predict prospective treatment for CDX based on our RNA-Seq data (see Supplementary Table S3 for the list of DE genes loaded to the PanDrugs database). Based on this analysis, we chose fostamatinib (SYK tyrosin kinase inhibitor) and vandetanib (VEGFR, EGFR, RET inhibitor) for therapeutic evaluation. As a reference, we utilised gemcitabine, a chemotherapy regimen which the patient received during her treatment before blood collection, and carboplatin (CBDCA), which was administered shortly after the blood collection (see Supplementary Fig. S1).

**Fig. 4:**
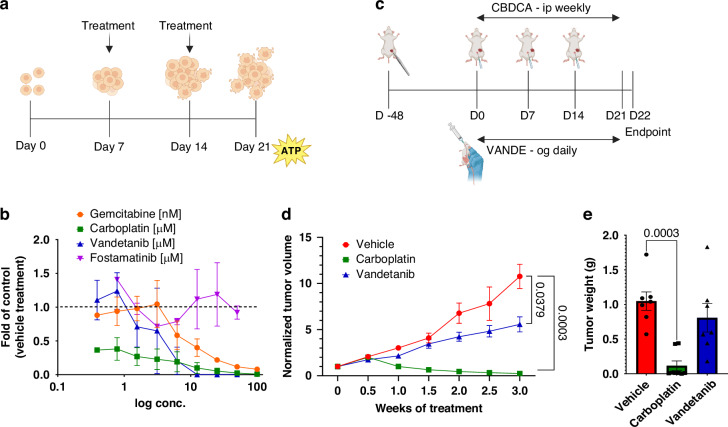
CTCs-derived xenograft and in vitro cultures are relevant models for drug testing. **a** Schematic workflow of drug treatment in in vitro 3D cell culture. Created in https://BioRender.com. **b** Analysis of spheroids’ viability at the endpoint using CellTitre Glo ATP assay. The data are presented as fold of control (vehicle treatment) at the endpoint (21 days), as a mean ± SEM from two independent repetitions performed in technical multiplicate. **c** Schematic workflow of mice treatment with carboplatin (CBDCA) and vandetanib (VANDE). Created in https://BioRender.com. **d** Normalised tumour volume after weekly intraperitoneal treatment with carboplatin (50 mg/kg), and daily treatment with vandetanib (25 mg/kg) via oral gavage for 21 days. Data represent mean ± SEM from two independent repetitions. **e** Tumour weight at the endpoint of the in vivo experiment. Data represent mean ± SEM from two independent repetitions. Statistical analysis was performed with the Mann-Whitney non-parametric test.

First, we treated the 3D cell culture with a range of concentrations for each selected drug (Fig. 4a, b and Supplementary Fig. S7A–C). The in vitro 3D cell culture demonstrated sensitivity to higher concentrations of gemcitabine and vandetanib, while fostamatinib treatment had minimal effect. Notably, the greatest sensitivity was observed with carboplatin treatment (Fig. 4b). In accordance with our results, when administered to the patient, gemcitabine treatment led to only a temporary clinical stabilisation and a slow laboratory elevation of CA15-3 (958 kU/l in June 2019 in the beginning of gemcitabine; 1215 kU/l in Aug 2019 when progression occurred). Next, we evaluated these findings in vivo using the CDX model. Based on the results obtained with in vitro cell culture, we tested the effect of carboplatin and vandetanib treatment on CDX growth in the mammary fat pad of immunodeficient mice (Fig. 4c). While treatment with carboplatin led to the complete suppression of tumour growth (Fig. 4d, e and Supplementary Fig. S7D, E), vandetanib treatment resulted in a moderate, statistically nonsignificant decrease in tumour volume and tumour weight. Examination of the lungs revealed disseminated cancer cells in vehicle-treated mice, whereas significantly fewer such cells were detected in the lungs of carboplatin-treated mice (Supplementary Fig. S7F and Supplementary Table S4). These results correspond with data obtained from an in vitro screen, where carboplatin treatment also displayed the most prominent effect. Together, despite RNA-Seq-guided drug selection, we identified carboplatin as the most effective treatment, showing strong tumour cell suppression both in vitro and in vivo, while fostamatinib had minimal impact and vandetanib exhibited only modest, non-significant effects. These findings highlight the predictive utility of the CDX models for preclinical therapeutic testing and potential personalised drug evaluation.

## Discussion

CTCs are regarded as mediators of primary tumour dissemination. Their analysis provides a unique insight into the process of tumour cell dissemination while also reflecting the biological state of both primary and secondary tumours. These findings significantly advance our understanding of the mechanisms underlying metastatic spread and offer a valuable foundation for the future identification of experimental biomarkers and therapeutic strategies. Therefore, the development of new models derived from CTCs, whether in vivo or in vitro, is of paramount importance. Here, we describe a unique in vivo xenograft and in vitro-derived cell culture established from circulating tumour cells of a patient diagnosed with multicentric invasive breast ductal carcinoma, which can serve as a valuable model for the metastatic stage of this disease.

The development of CTC-derived experimental models possesses several obstacles that must be critically evaluated. One of the crucial factors with significant influence is the method of CTCs isolation (summarised in [3]). Knowing the disadvantages of marker-based selection, we chose the negative depletion of CD45^+^ cells in combination with gradient centrifugation in our study. This methodological approach does not result in the isolation of a pure population of CTCs, but rather in obtaining a CTC-enriched population depleted of most CD45^+^ cells. The derivation of in vivo models from CTCs has faced variable, but generally very low efficiency (summarised in [9]). In our project, we collected blood from 86 metastatic BCa patients between November 2019 and December 2023. We have been focused on the careful optimisation of each step during CDX generation, including CTCs isolation using filtration, isolation from a larger volume of blood, and subcutaneous, mammary fat pad, caudal artery, or subrenal capsule implantation. Despite this effort, we obtained only two CTC-derived xenografts from the blood of patients diagnosed with metastatic BCa (success rate 2.3%). The latency from implantation to isolation of the xenograft described here was 175 days, and the latency of the second xenograft was 287 days. It is important to note that although we microscopically validated the presence of CTCs in the enriched fraction after completing all steps of the blood processing, the exact number of CTCs was not determined before implantation in mice. The reason for this was to maximise the number of implanted CTCs, because it is known that the number of initially implanted CTCs seems to be important for CDX generation, since CDXs were usually generated from patients with higher CTC counts, as described, for example, for small cell lung cancer-derived CDX [35] or prostate cancer-derived CDX [36]. We are aware that the absence of this information is one of the limitations of our study. On the other hand, our effort was to establish a CDX model rather than investigating the number of CTCs required for the successful generation of CDX.

The long latency and lack of standardisation are significant barriers to using CDX models in precision medicine for testing treatment options. This can be overcome by establishing CTC-derived cell cultures in vitro. Numerous studies describe the establishment of short-term or long-term BCa CTC-derived in vitro cultures [37–40], which takes several weeks, not months, as in the case of xenografts. However, there is no standardised workflow for in vitro expansion, and various culture conditions or cultivation media are used in each study. Only recently, Würth and colleagues described a platform for the generation of CTC-derived organoids in vitro in specific cultivation media containing neuregulin-1, which allows long-term expansion of CTCs from multiple patients with different subtypes of metastatic breast cancer [41]. In another recent study, CTCs from gastroesophageal cancer were expanded in vitro in a co-culture system with peripheral blood mononuclear cells from the same patient, with a success rate of 66% [42]. Here, we took advantage of a published protocol for in vitro expansion of patient-derived xenografts [17] and established an in vitro cell culture from the human cancer cells sorted from CDX_IBP_01; this cell culture retained its tumorigenic potential. For further application, the phenotypic stability of both in vitro and in vivo models is crucial. Therefore, we profiled xenograft and in vitro cell cultures with a panel of surface markers associated with the epithelial and mesenchymal phenotype of breast cancer cells [43]. Both in vitro cell cultures and CDX were entirely positive for epithelial markers EpCAM and CD9; all other epithelial and mesenchymal markers were variably expressed in all tested conditions, with mesenchymal markers being expressed more frequently by in vitro cell cultures in general. Next, we uncovered that while cancer cells from the original CDX were almost entirely CD44^−^/CD24^+^, expansion in vitro led to a phenotypical shift with significantly more cells expressing the stem-like cell phenotype CD44^+^/CD24^−^. We assume that an increase in the subpopulation of CSCs and a more mesenchymal phenotype is associated with adaptation to in vitro conditions. Liu and colleagues used the syngeneic murine 4T1 model to describe the presence of inter-and intraindividual EMT heterogeneity in CTCs and the co-existence of CTCs with epithelial and mesenchymal phenotype in the same animal [44]. Since we were not able to characterise the original CTCs from the patient, we profiled primary CDX tumour–CTCs in the blood of tumour-bearing animals and disseminated cells (DTCs) of these animals. Using the detection of surface Trop-2 as a marker of cancer cell plasticity [31], we showed that the majority of human cancer cells in the primary tumour and disseminated tumour cells in lungs are positive for this marker, while circulating tumour cells in blood are heterogeneous in terms of Trop-2 expression, as a Trop-2-negative subpopulation is also present in the blood. These results confirm that our model is metastatic and can be used for the study of phenotypic plasticity of circulating tumour cells and disseminated tumour cells.

A comparison of the characteristics of CTCs, or CTC-derived models, with those of primary tumours reveals changes and adaptive mechanisms crucial for metastasis development. During their travel in circulation, CTCs exhibit significant metabolic plasticity, allowing them to adapt to the harsh conditions in the bloodstream. They reprogramme their metabolism to support energy needs, resistance to oxidative stress, and reactive oxygen species (ROS) detoxification. This is achieved by enhancing the antioxidant metabolism, in particular, upregulation of NAD^+^ kinase (NADK), the enzyme responsible for de novo synthesis of NADP(H), allowing for effective antioxidant response [45, 46]. Indeed, our transcriptome profiling comparing the primary tumour and CDX revealed profound changes between these two models. Namely, genes associated with mitochondrial function, mass, and/or fitness were significantly upregulated in the CDX. This is in consistence with a previous study which found increased oxidative phosphorylation and mitochondrial biogenesis in CTCs with respect to the primary tumour in a mouse model using 4T1 mouse BCa cells [47]. Next, transcriptomic analysis revealed the upregulation of genes associated with cell division, proliferation, and cell cycle progression, as well as the downregulation of genes linked to EMT, extracellular matrix interaction, and focal adhesions. These data indicate the complex transformation of cancer cells into CTCs that can successfully develop metastases. On the other hand, since we did not analyse the patient’s CTCs, we cannot rule out the possibility that observed changes are related to the adaptation of the patient´s CTCs to growth in vivo in mice. Furthermore, we are fully aware that our CDX model was derived from CTCs of a patient with metastasis, who has undergone numerous cycles of treatment. Therefore, the observed differences may also be attributed to this heavy treatment rather than metastatic transformation.

A common motivation for the development and refinement of CTC-derived models is their use for finding personalised treatment for individual patients. Therefore, we performed a small preclinical study to demonstrate the usability of our model for drug treatment screening. Firstly, we tested selected drugs in vitro. We chose gemcitabine, which the patient received previously, and carboplatin, which the patient received shortly after the blood collection for CTCs isolation. Next, based on our RNA-Seq data and the PanDrugs online tool [33, 34], we tested vandetanib (Caprelsa), used for the treatment of medullary thyroid cancer [48], and fostamatinib (Tavalisse), used for the treatment of chronic immune thrombocytopenia [49], as drugs predicted to be effective in our model. In vitro results show that carboplatin treatment was the most effective in inhibiting the growth of spheroids. When moving to in vivo studies, similar results were obtained, as carboplatin treatment led to a significant reduction in tumour volume. A similar trend of reduced tumour growth was observed with vandetanib treatment in vivo. Although not primarily dedicated to the treatment of breast cancer, several studies indicate that vandetanib, a VEGFR-2, EGFR, and RET inhibitor, might be effective against breast cancer, especially in combination with other treatments [50–52]. We are aware that our mouse cohort is small, and we tested only one concentration and one scheme of administration to mice; therefore, the possibility of generalising the obtained results is quite limited. A previous study using a patient-derived metastatic xenograft model of human gastric cancer showed that monitoring the number and phenotype of CTCs can serve as an effective indicator of the efficacy of anticancer therapy [53]. Although we did not perform phenotypic characterisation of CTCs in response to drug treatment, we quantified disseminated cancer cells in lungs and found that carboplatin and, to a lesser extent, vandetanib, reduced the number of cancer cells disseminated in the lungs in our model.

The patient´s treatment with carboplatin (CBDCA) was clinically well tolerated, regression of supraclavicular lymph nodes was observed after the first dose of CBDCA, and the patient felt better. Laboratory results showed normalisation of CRP (32 mg/L to 8 mg/L) and a decrease in CA 15-3. The value of CA 15-3 was 4097 kU/L at the time of CBDCA initiation. After the third cycle of CBDCA, it was 3106 kU/L. In April, after 6 cycles, and at the time of diagnosis of brain metastasis, it was 1454 kU/L. The CBDCA dose had to be gradually reduced due to thrombocytopenia. CBDCA penetration into the CNS is limited by the blood-brain barrier, and this may be one of the reasons for the progression in the CNS, even with clinical improvement and decreasing CA 15-3. Another reason for progression may be the lower dose of CBDCA, limited by thrombocytopenia, which is the most common type of toxicity, especially in heavily pre-treated patients [54, 55].

We are aware that a significant limitation of our study is the absence of CTC characterisation similar to that we performed for the primary tumour and established CDX characterisation. However, we believe that the xenotransplant and derived in vitro cultures presented here will be recognised as valid models for studying advanced stages of breast cancer and drug efficacy.

## Conclusions

Overall, here we describe a CTC-derived in vivo and in vitro model for advanced BCa, confirming both the tumorigenic and metastatic potential of original CTCs, which may be usable in future studies of BCa dissemination, circulating tumour cell plasticity, and as a model for testing the effect of newly proposed treatments.

## Supplementary information

Supplementary Figure S1

Supplementary Figure S2

Supplementary Figure S3

Supplementary Figure S4

Supplementary Figure S5

Supplementary Figure S6

Supplementary Figure S7

Supplementary Table S1

Supplementary Table S2

Supplementary Table S3

Supplementary Table S4

Supplementary Table S5

Supplementary Figure legends

## Data Availability

The datasets generated and used in the current study are available from the corresponding author on reasonable request.
