# Supplementary figures and images for: Circulating tumour cell-derived xenograft as a preclinical platform for metastatic breast cancer
Source: Br J Cancer. 2026 May 18;135(4):568–80. doi: 10.1038/s41416-026-03468-0 (PMC13427727; doi:10.1038/s41416-026-03468-0)

A

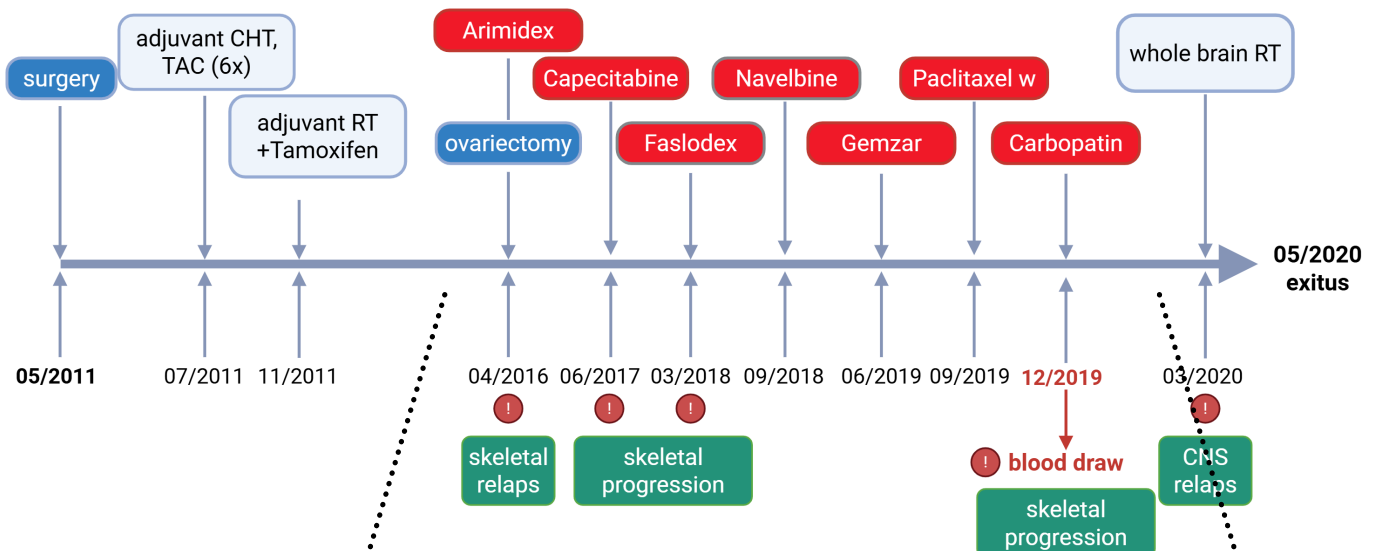

B

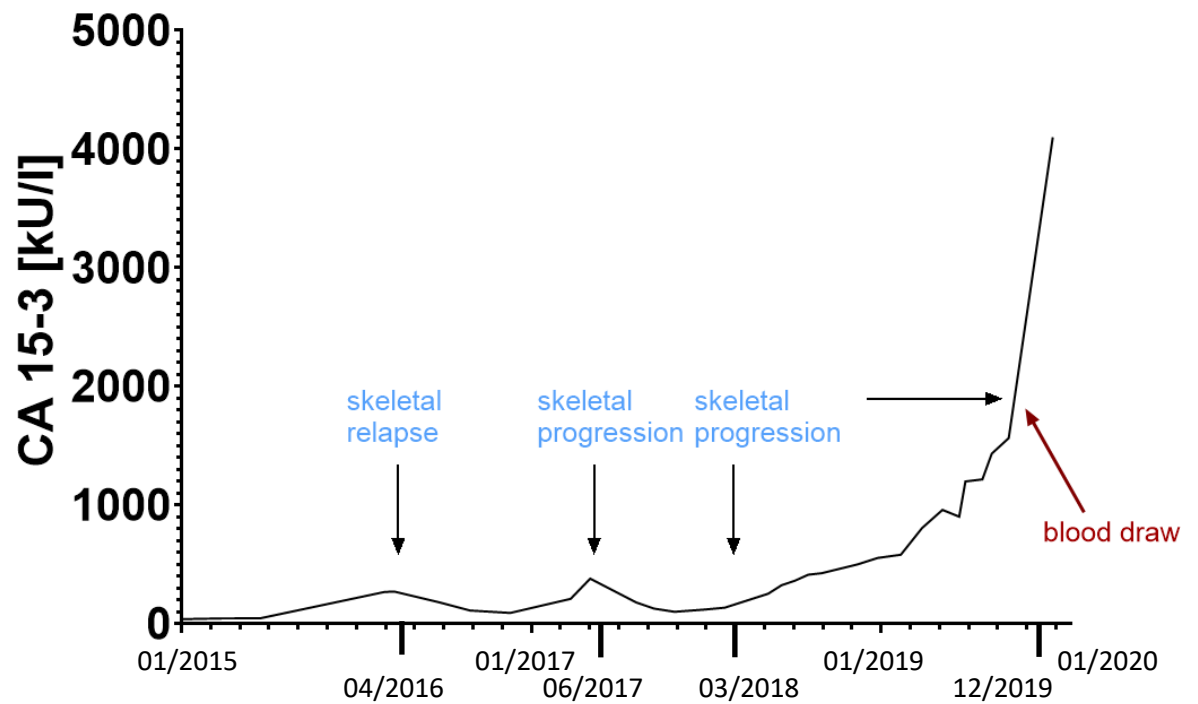

C

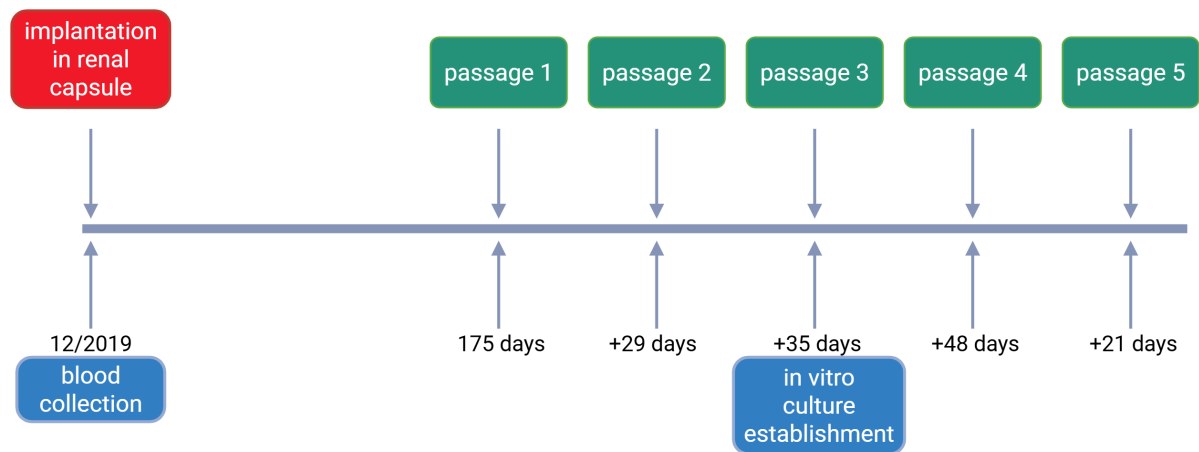

Supplement: Supplementary file 1 — Supplementary Figure S1 [file 41416_2026_3468_MOESM1_ESM.pdf]

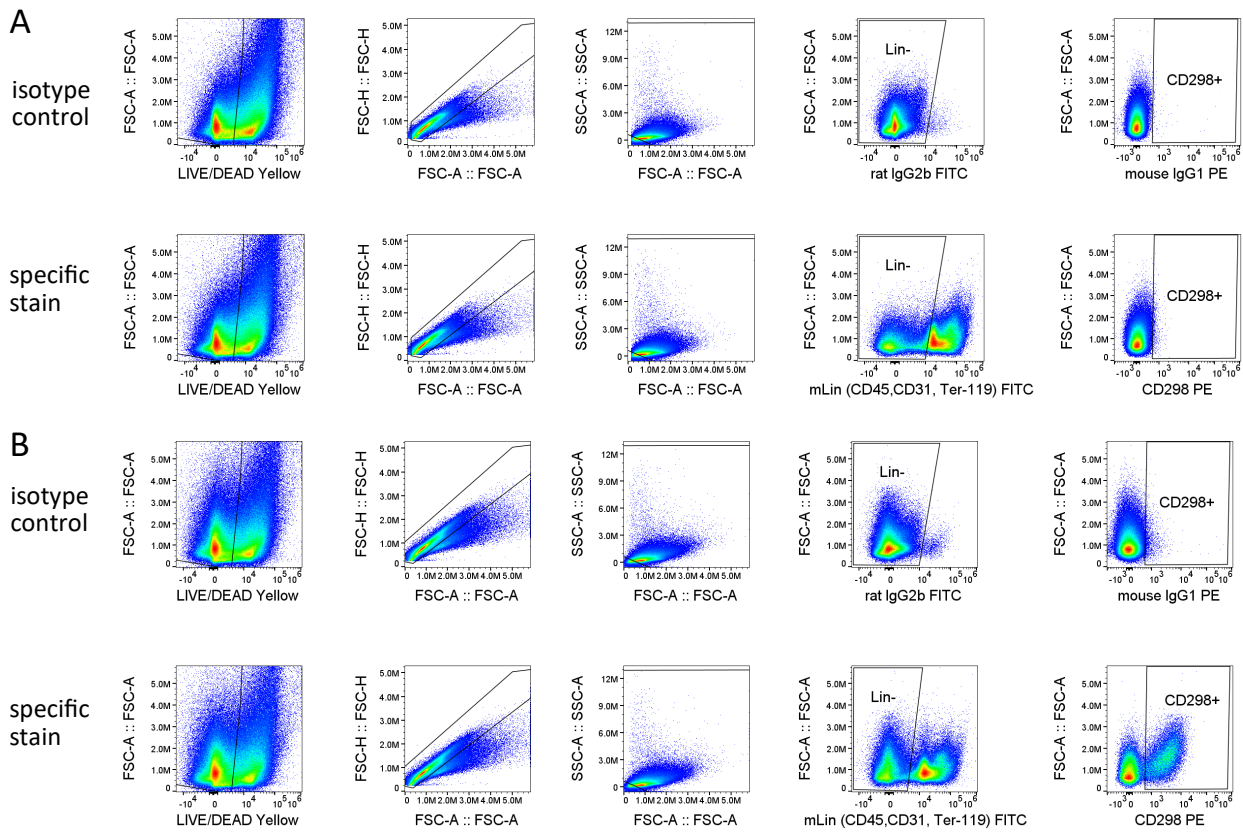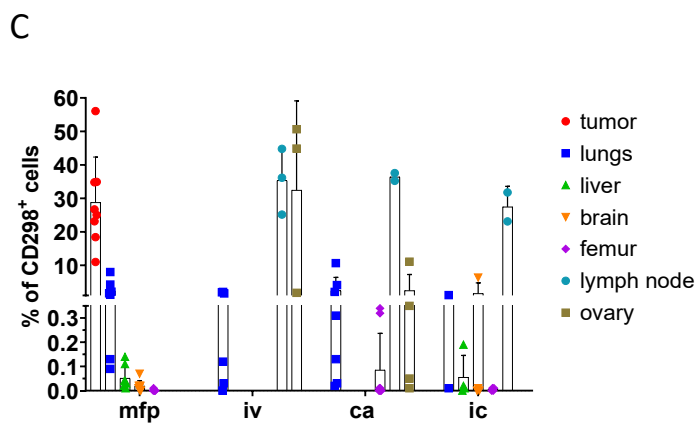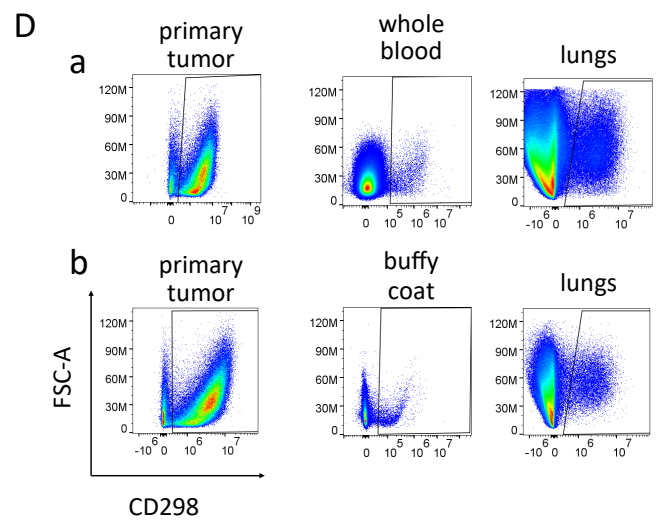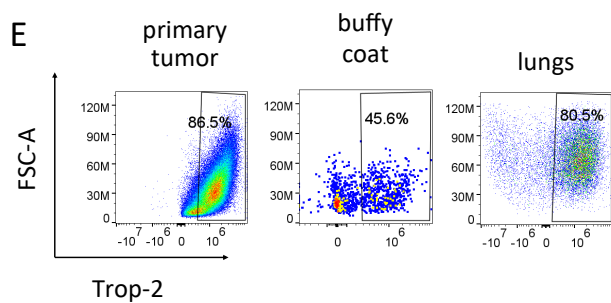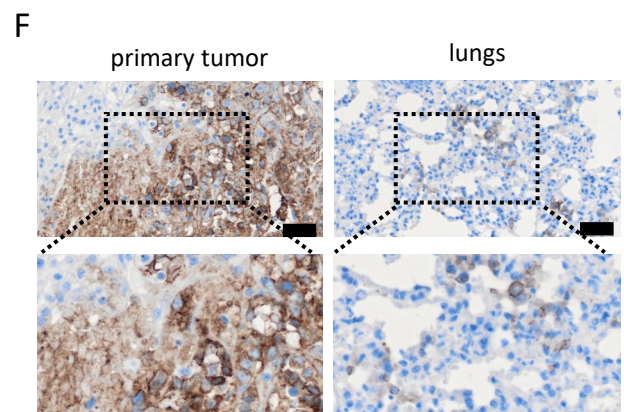

Supplement: Supplementary file 2 — Supplementary Figure S2 [file 41416_2026_3468_MOESM2_ESM.pdf]

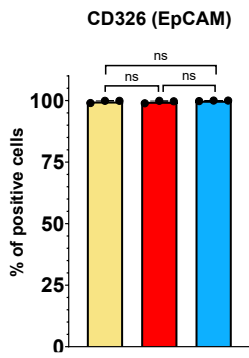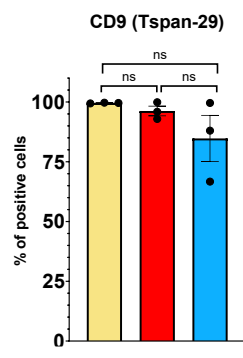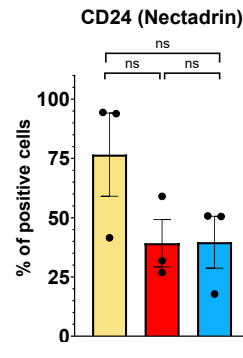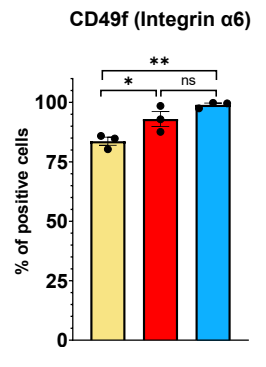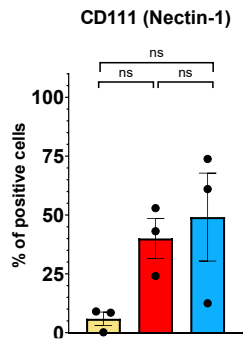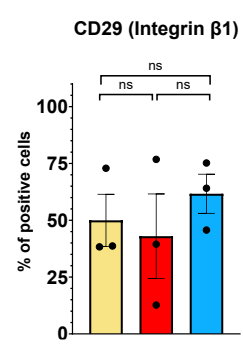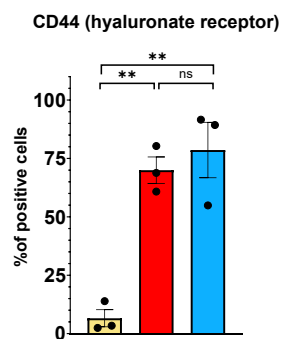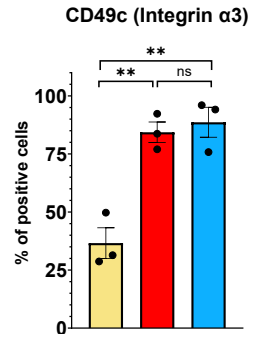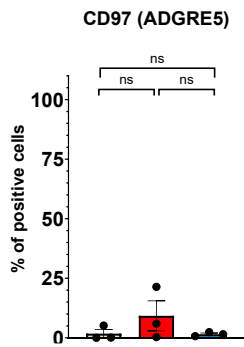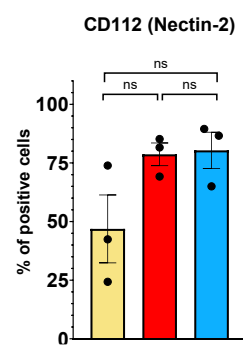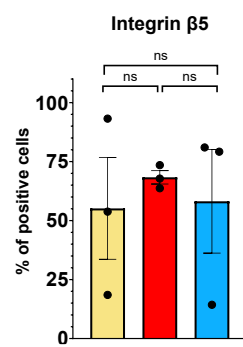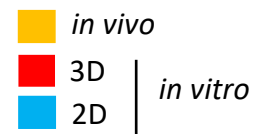

Supplement: Supplementary file 3 — Supplementary Figure S3 [file 41416_2026_3468_MOESM3_ESM.pdf]

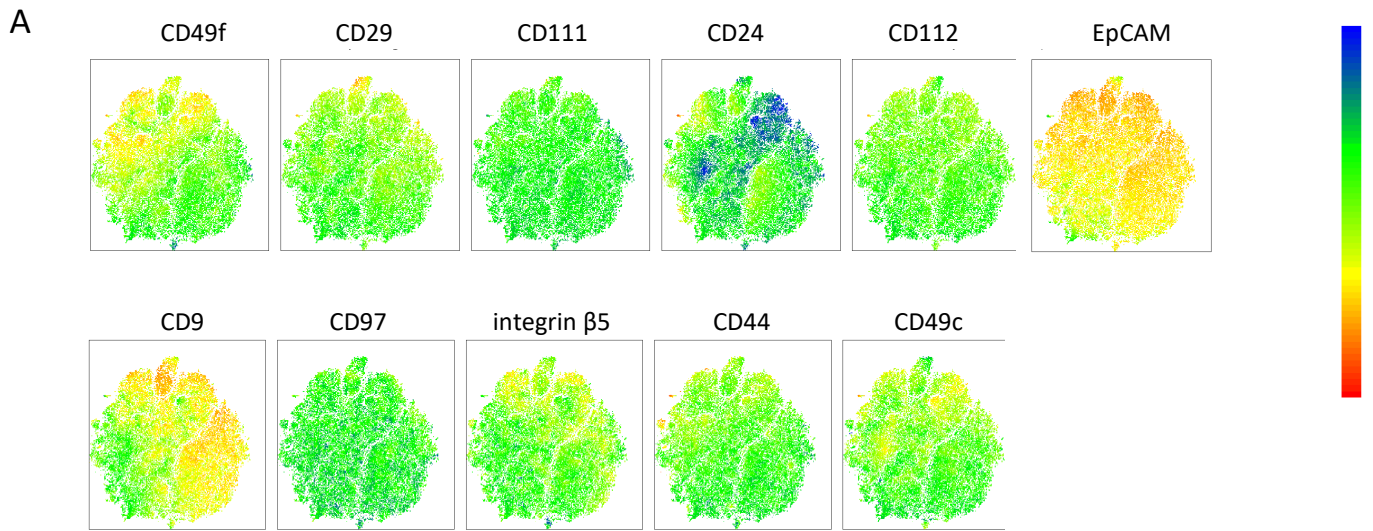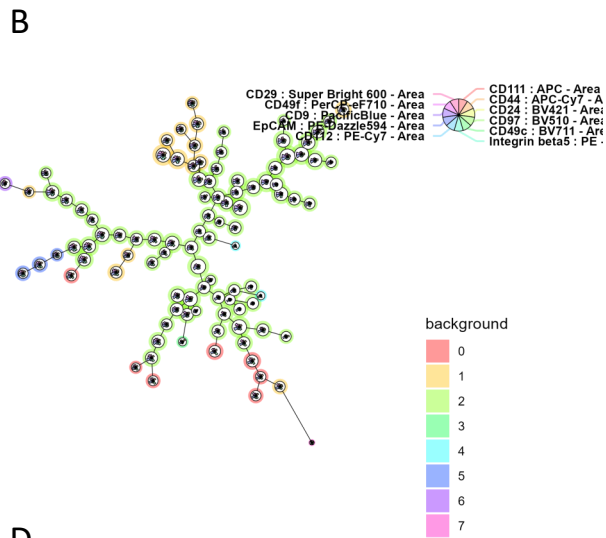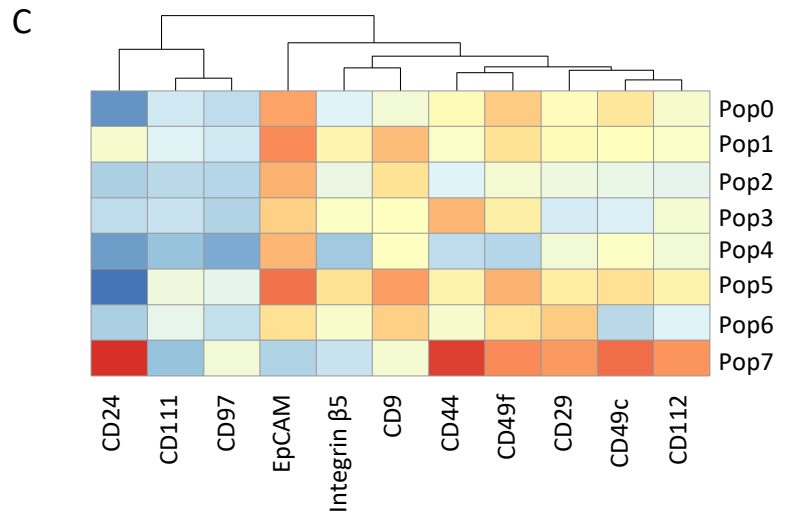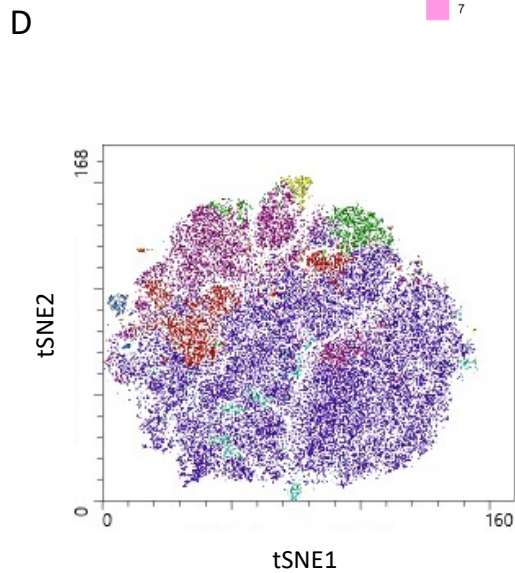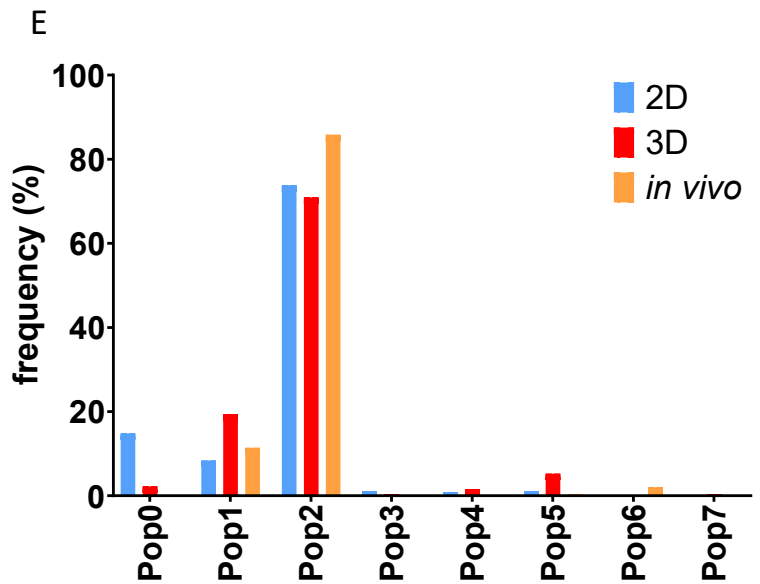

Supplement: Supplementary file 4 — Supplementary Figure S4 [file 41416_2026_3468_MOESM4_ESM.pdf]

**A**

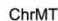

Position on chromosome (Mbp)

B

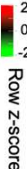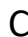

D

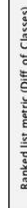

# E

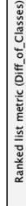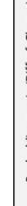

Supplement: Supplementary file 6 — Supplementary Figure S6 [file 41416_2026_3468_MOESM6_ESM.pdf]

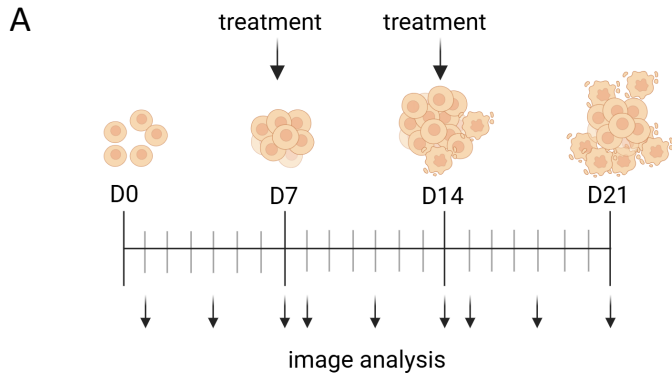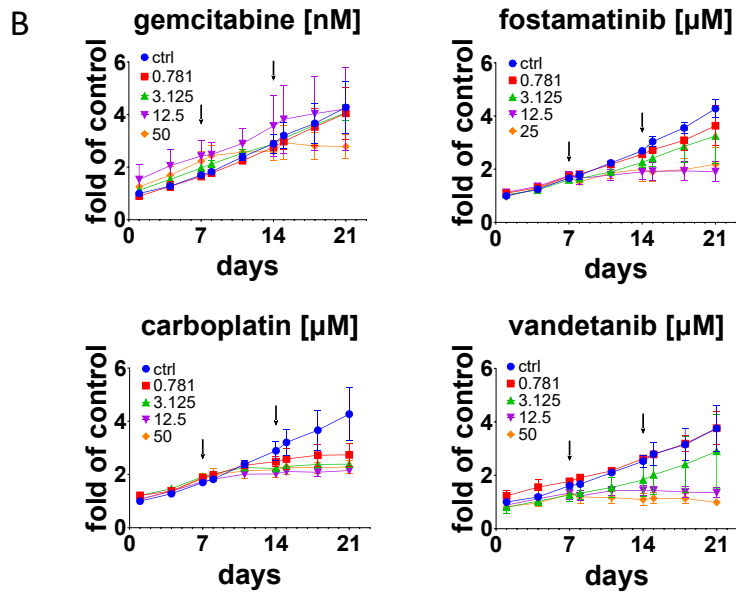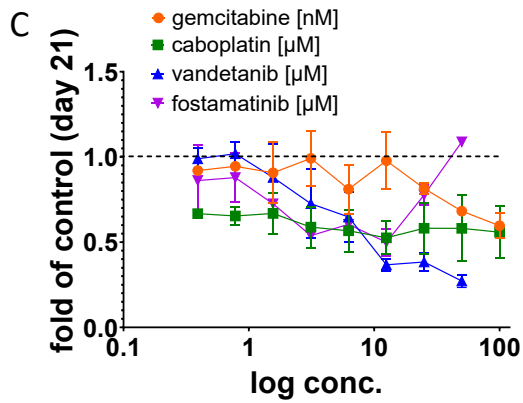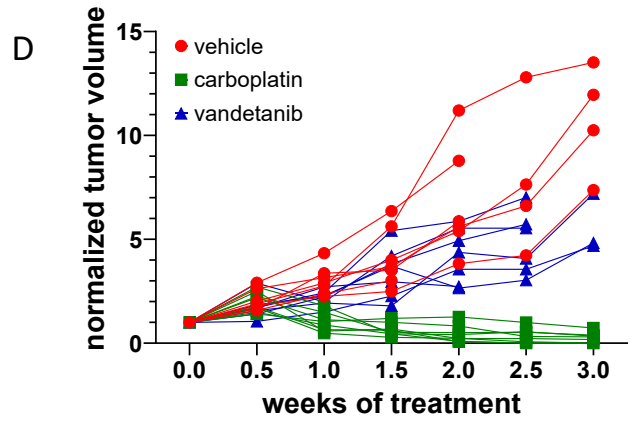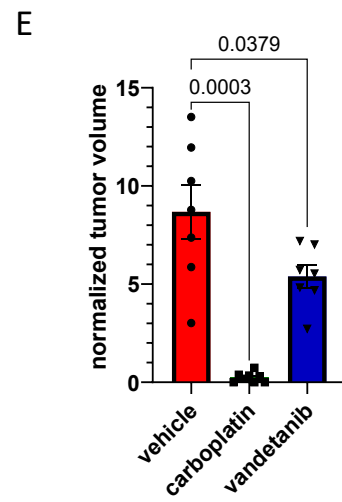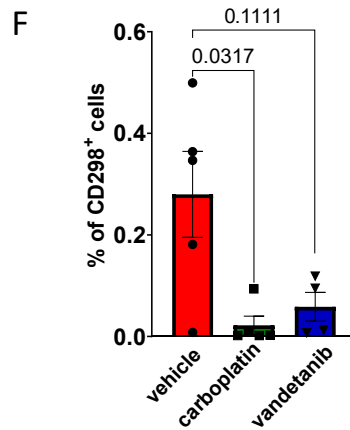

Supplement: Supplementary file 7 — Supplementary Figure S7 [file 41416_2026_3468_MOESM7_ESM.pdf]
