# Supplementary material for: Circulating tumour cell-derived xenograft as a preclinical platform for metastatic breast cancer
Source: Br J Cancer. 2026 May 18;135(4):568–80. doi: 10.1038/s41416-026-03468-0 (PMC13427727; doi:10.1038/s41416-026-03468-0)
Supplement: Supplementary file 5 — Supplementary Figure S5 [file 41416_2026_3468_MOESM5_ESM.pdf]

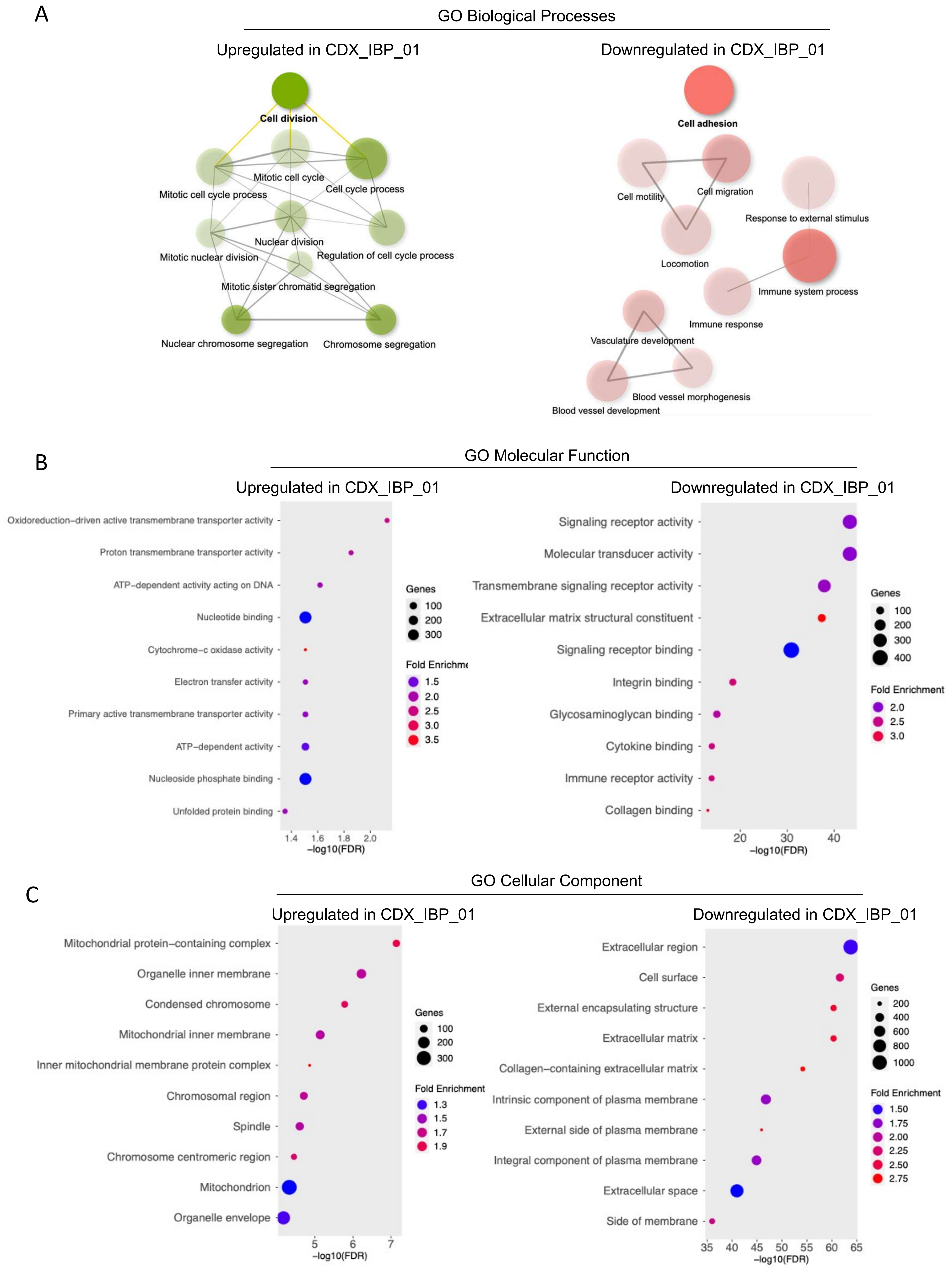

A
GO Biological Processes
Upregulated in CDX_IBP_01
Cell division
Mitotic cell cycle
Cell cycle process
Mitotic cell cycle process
Nuclear division
Mitotic nuclear division
Regulation of cell cycle process
Mitotic sister chromatid segregation
Nuclear chromosome segregation
Chromosome segregation
Downregulated in CDX_IBP_01
Cell adhesion
Cell motility
Cell migration
Response to external stimulus
Locomotion
Immune system process
Immune response
Vasculature development
Blood vessel morphogenesis
Blood vessel development
B
GO Molecular Function
Upregulated in CDX_IBP_01
Oxidoreduction−driven active transmembrane transporter activity
Proton transmembrane transporter activity
ATP−dependent activity acting on DNA
Nucleotide binding
Cytochrome−c oxidase activity
Electron transfer activity
Primary active transmembrane transporter activity
ATP−dependent activity
Nucleoside phosphate binding
Unfolded protein binding
1.4 1.6 1.8 2.0
−log10(FDR)
Genes
100
200
300
Fold Enrichme
1.5
2.0
2.5
3.0
3.5
Downregulated in CDX_IBP_01
Signaling receptor activity
Molecular transducer activity
Transmembrane signaling receptor activity
Extracellular matrix structural constituent
Signaling receptor binding
Integrin binding
Glycosaminoglycan binding
Cytokine binding
Immune receptor activity
Collagen binding
20 30 40
−log10(FDR)
Genes
100
200
300
400
Fold Enrichment
2.0
2.5
3.0
C
GO Cellular Component
Upregulated in CDX_IBP_01
Mitochondrial protein−containing complex
Organelle inner membrane
Condensed chromosome
Mitochondrial inner membrane
Inner mitochondrial membrane protein complex
Chromosomal region
Spindle
Chromosome centromeric region
Mitochondrion
Organelle envelope
5 6 7
−log10(FDR)
Genes
100
200
300
Fold Enrichment
1.3
1.5
1.7
1.9
Downregulated in CDX_IBP_01
Extracellular region
Cell surface
External encapsulating structure
Extracellular matrix
Collagen−containing extracellular matrix
Intrinsic component of plasma membrane
External side of plasma membrane
Integral component of plasma membrane
Extracellular space
Side of membrane
35 40 45 50 55 60 65
−log10(FDR)
Genes
200
400
600
800
1000
Fold Enrichment
1.50
1.75
2.00
2.25
2.50
2.75
