# Supplementary material for: Circulating tumour cell-derived xenograft as a preclinical platform for metastatic breast cancer
Source: Br J Cancer. 2026 May 18;135(4):568–80. doi: 10.1038/s41416-026-03468-0 (PMC13427727; doi:10.1038/s41416-026-03468-0)
Supplement: Supplementary file 8 — Supplementary Table S1 [file 41416_2026_3468_MOESM8_ESM.docx]

**Supplementary Table S1. List of antibodies, isotype controls, and dead cell stain reagents used for flow cytometry.**

| **marker** | **conjugate** | **vendor** | **catalog number** | **dilution** |
| --- | --- | --- | --- | --- |
| **Primary antibodies** | | | | |
| CD44 (hyaluronon receptor) | APC-Cy7 | SONY | 1115140 | 1:100 |
| CD49f (Integrin α6) | PerCP-eFluor710 | eBioscience | 46-0495-82 | 1:333 |
| CD112 (Nectin-2) | PE-Cy7 | BioLegend | 337414 | 1:100 |
| CD9 (Tspan-29) | Pacific Blue | Exbio | PB-208-T100 | 1:20 |
| CD24 (Nectadrin) | BV421 | SONY | 2155610 | 1:400 |
| CD326 (EpCAM) | PE-Dazzle 594 | SONY | 2221160 | 1:400 |
| CD29 (Integrin β1) | SuperBright 600 | Thermo Fisher Scientific | 63-0299-42 | 1:20 |
| CD111 (Nectin-1) | APC | Miltenyi Biotec | 130-103-834 | 1:11 |
| CD97 (ADGRE5) | Briliant Violet 510 | BD Bioscience | 742446 | 1:50 |
| Integrin β5 | PE | Biolegend 345204 | 345204 | 1:200 |
| CD49c (Integrin α3) | Briliant Violet 711 | BD Bioscience | 744520 | 1:400 |
| CD298 | FITC | Miltenyi Biotec | 130-123-234 | 1:100 |
| CD298 | PE | BioLegend | 341704 | 1:50 |
| CD45 | FITC | SONY | 1338550 | 1:250 |
| CD31 | FITC | SONY | 1112030 | 1:250 |
| Ter-119 | FITC | SONY | 1181030 | 1:250 |
| **Isotype controls** | | | | |
| rat IgG2a,kappa | APC-Cy7 | SONY | 2603120 | 1:100 |
| rat IgG2a,kappa | PeCP-eFlour 710 | eBioschience | 46-4321 | 1:333 |
| Mouse IgG1 | PE-Cy7 | Biolegend | 400126 | 1:100 |
| Mouse IgG1 | Pacific Blue | Exbio | PB-632-C100 | 1:40 |
| Mouse IgGa kappa | Brilian Violet 421 | SONY | 2601300 | 1:400 |
| Mouse IgGb, kappa | PE-Dazzle 594 | SONY | 2601790 | 1:400 |
| Mouse IgGa,kappa | SuperBright 600 | ThermoFisher Scientific | 63-4714-82 | 1:20 |
| Mouse IgG1 | APC | Miltenyi Biotec | 130-092-214 | 1:11 |
| Mouse IgG2a, kappa | Briliant Violet 510 | SONY | 2601340 | 1:25 |
| mouse IgG1 | PE | Biolegend | 400112 | 1:71 |
| Mouse IgG2a | PE | BioLegend | 400212 | 1:250 |
| Mouse IgG1, kappa | Briliant Violet 711 | BD Bioscience | 563044 | 1:400 |
| REA control | FITC | Miltenyi Biotec | 130-104-610 | 1:100 |
| Mouse IgG1 | PE | BioLegend | 400112 | 1:71,5 |
| Rat IgG2b | FITC | SONY | 2603030 | 3:250 |
| **LIVE/DEAD® Fixable DEAD CELL STAIN KITs** | | | | |
| Yellow |  | ThermoFisher Scientific | L34959 | 1:500 |
| Far Red |  | ThermoFisher Scientific | L10120 | 1:1000 |
| Aqua |  | ThermoFisher Scientific | L34966 | 1:500 |
