# Supplementary material for: Circulating tumour cell-derived xenograft as a preclinical platform for metastatic breast cancer
Source: Br J Cancer. 2026 May 18;135(4):568–80. doi: 10.1038/s41416-026-03468-0 (PMC13427727; doi:10.1038/s41416-026-03468-0)
Supplement: Supplementary file 9 — Supplementary Table S2 [file 41416_2026_3468_MOESM9_ESM.docx]

**Supplementary Table S2. Table summarizing number of all analysed events vs. the number of CD298^+^ cells from each organ of each mouse analysed in the metastatic in vivo assay. Related to Figure 1D.**

|  | | **tumor** | | | **lungs** | | | **liver** | | | **brain** | | | **femur** | | | **lymph node** | | | **ovary** | | |
| --- | --- | --- | --- | --- | --- | --- | --- | --- | --- | --- | --- | --- | --- | --- | --- | --- | --- | --- | --- | --- | --- | --- |
|  |  | **live events** | **CD298+** | **% of live** | **live events** | **CD298+** | **% of live** | **live events** | **CD298+** | **% of live** | **live events** | **CD298+** | **% of live** | **live events** | **CD298+** | **% of live** | **live events** | **CD298+** | **% of live** | **live events** | **CD298+** | **% of live** |
| **mfp 1** | 4973 | 512397 | 136820 | 26,70 | 338443 | 4027 | 1,19 | 473650 | 149 | 0,03 | 476900 | 3 | 0,00 | 312992 | 7 | 0,00 | na | | | na | | |
|  | 4970 | 314255 | 109703 | 34,91 | 261287 | 235 | 0,09 | 403871 | 89 | 0,02 | 482479 | 112 | 0,02 | 381438 | 20 | 0,01 | na | | | na | | |
| **mfp 2** | 783 | 236554 | 54764 | 23,15 | 279256 | 22378 | 8,01 | 248028 | 92 | 0,04 | 26294 | 5 | 0,02 | 132901 | 2 | 0,00 | na | | | na | | |
|  | 784 | 284863 | 71339 | 25,04 | 318978 | 2813 | 0,88 | 195358 | 31 | 0,02 | 315207 | 47 | 0,01 | 393462 | 0 | 0,00 | na | | | na | | |
|  | 786 | 255328 | 47023 | 18,42 | 352605 | 459 | 0,13 | 186106 | 22 | 0,01 | 192120 | 1 | 0,00 | 314585 | 1 | 0,00 | na | | | na | | |
| **mfp 3** | 742 | 268969 | 29590 | 11,00 | 325037 | 5270 | 1,62 | 160448 | 169 | 0,11 | 27556 | 6 | 0,02 | 367167 | 3 | 0,00 | na | | | na | | |
|  | 743 | 135752 | 47252 | 34,81 | 130002 | 5556 | 4,27 | 167620 | 235 | 0,14 | 30549 | 20 | 0,07 | 260095 | 4 | 0,00 | na | | | na | | |
|  | 744 | 208797 | 117074 | 56,07 | 289220 | 6197 | 2,14 | 235251 | 89 | 0,04 | 79783 | 5 | 0,01 | 199573 | 0 | 0,00 | na | | | na | | |
|  | | | | | | | | | | | | | | | | | | | | | | |
|  | | **tumor** | | | **lungs** | | | **liver** | | | **brain** | | | **femur** | | | **lymph node** | | | **ovary** | | |
|  |  | **live events** | **CD298+** | **% of live** | **live events** | **CD298+** | **% of live** | **live events** | **CD298+** | **% of live** | **live events** | **CD298+** | **% of live** | **live events** | **CD298+** | **% of live** | **live events** | **CD298+** | **% of live** | **live events** | **CD298+** | **% of live** |
| **tail vein 1** | 4971 | na | | | 94357 | 1953 | 2,07 | na | | | na | | | na | | | 85367 | 30866 | 36,16 | na | | |
|  | 4969 | na | | | 74029 | 279 | 0,38 | na | | | na | | | na | | | na | | | na | | |
| **tail vein 2** | 791 | na | | | 355419 | 6865 | 1,93 | na | | | na | | | na | | | 266885 | 119536 | 44,79 | 222457 | 99798 | 44,86 |
|  | 705 | na | | | 397504 | 6313 | 1,59 | na | | | na | | | na | | | 304983 | 76694 | 25,15 | 386760 | 195876 | 50,65 |
|  | 788 | na | | | 335003 | 1653 | 0,49 | na | | | na | | | na | | | na | | | 9089 | 162 | 1,78 |
| **tail vein 3** | 730 | na | | | 80182 | 0 | 0,00 | na | | | na | | | na | | | na | | | na | | |
|  | 733 | na | | | 258753 | 316 | 0,12 | na | | | na | | | na | | | na | | | na | | |
|  | 731 | na | | | 97425 | 26 | 0,03 | na | | | na | | | na | | | na | | | na | | |
|  | | | | | | | | | | | | | | | | | | | | | | |
|  | | **tumor** | | | **lungs** | | | **liver** | | | **brain** | | | **femur** | | | **lymph node** | | | **ovary** | | |
|  |  | **live events** | **CD298+** | **% of live** | **live events** | **CD298+** | **% of live** | **live events** | **CD298+** | **% of live** | **live events** | **CD298+** | **% of live** | **live events** | **CD298+** | **% of live** | **live events** | **CD298+** | **% of live** | **live events** | **CD298+** | **% of live** |
| **caudal artery 1** | 4972 | na | | | na | | | na | | | na | | | 169440 | 14 | 0,01 | na | | | 13287 | 7 | 0,05 |
|  | 4974 | na | | | 156973 | 16739 | 10,66 | na | | | na | | | 147075 | 506 | 0,34 | 207630 | 73125 | 35,22 | na | | |
| **caudal artery 2** | 789 | na | | | 341843 | 14004 | 4,10 | na | | | na | | | 269417 | 850 | 0,32 | 328481 | 123303 | 37,54 | 328481 | 36396 | 11,08 |
|  | 790 | na | | | 360222 | 72 | 0,02 | na | | | na | | | 416510 | 6 | 0,00 | na | | | 3397 | 12 | 0,35 |
|  | 792 | na | | | 354310 | 90 | 0,03 | na | | | na | | | 450015 | 6 | 0,00 | na | | | 7386 | 1 | 0,01 |
| **caudal artery 3** | 732 | na | | | 174914 | 224 | 0,13 | na | | | na | | | 395413 | 0 | 0,00 | na | | | na | | |
|  | 729 | na | | | 80182 | 1656 | 2,07 | na | | | na | | | 398901 | 2 | 0,00 | na | | | 6701 | 42 | 0,63 |
|  | 734 | na | | | 212016 | 662 | 0,31 | na | | | na | | | 475171 | 25 | 0,01 | na | | | na | | |
|  | | | | | | | | | | | | | | | | | | | | | | |
|  | | **tumor** | | | **lungs** | | | **liver** | | | **brain** | | | **femur** | | | **lymph node** | | | **ovary** | | |
|  |  | **live events** | **CD298+** | **% of live** | **live events** | **CD298+** | **% of live** | **live events** | **CD298+** | **% of live** | **live events** | **CD298+** | **% of live** | **live events** | **CD298+** | **% of live** | **live events** | **CD298+** | **% of live** | **live events** | **CD298+** | **% of live** |
| **intracardial 2** | 782 | na | | | 214797 | 2379 | 1,11 | 28382 | 53 | 0,19 | 264400 | 19 | 0,01 | 448446 | 7 | 0,00 | 517287 | 119545 | 23,11 | na | | |
|  | 787 | na | | | 329793 | 2718 | 0,82 | 282599 | 57 | 0,02 | 91946 | 5809 | 6,32 | 135436 | 9 | 0,01 | 284604 | 90452 | 31,78 | na | | |
| **intracardiac 3** | 741 | na | | | 258511 | 33 | 0,01 | 110948 | 3 | 0,00 | 263148 | 1 | 0,00 | 418207 | 3 | 0,00 | na | | | na | | |
|  | 746 | na | | | 191945 | 1065 | 0,55 | 86284 | 8 | 0,01 | 42974 | 2 | 0,00 | 375732 | 49 | 0,01 | na | | | na | | |
