# Supplementary material for: Circulating tumour cell-derived xenograft as a preclinical platform for metastatic breast cancer
Source: Br J Cancer. 2026 May 18;135(4):568–80. doi: 10.1038/s41416-026-03468-0 (PMC13427727; doi:10.1038/s41416-026-03468-0)
Supplement: Supplementary file 11 — Supplementary Table S4 [file 41416_2026_3468_MOESM11_ESM.docx]

**Supplementary Table S4. Percentage of CD298+ cells from live cells related to Supplementary Figure S7F.**

| **group** | **mouse** | **live cells** | **CD298+** | **% of CD298^+^ from live cells** |
| --- | --- | --- | --- | --- |
| **control** | 1467 | 273880 | 22 | 0,008 |
|  | 1442 | 575000 | 2092 | 0,364 |
|  | 1443 | 493000 | 1709 | 0,347 |
|  | 1429 | 493000 | 893 | 0,181 |
|  | 1431 | 358513 | 1790 | 0,499 |
| **carboplatin** | 1464 | 429000 | 402 | 0,094 |
|  | 1466 | 353175 | 9 | 0,003 |
|  | 1446 | 402000 | 7 | 0,002 |
|  | 1447 | 370473 | 7 | 0,002 |
|  | 1428 | 402000 | 41 | 0,010 |
| **vandetanib** | 1463 | 395299 | 50 | 0,013 |
|  | 1427 | 487000 | 35 | 0,007 |
|  | 1430 | 357825 | 340 | 0,095 |
|  | 1441 | 476000 | 565 | 0,119 |
