# Supplementary material for: Circulating tumour cell-derived xenograft as a preclinical platform for metastatic breast cancer
Source: Br J Cancer. 2026 May 18;135(4):568–80. doi: 10.1038/s41416-026-03468-0 (PMC13427727; doi:10.1038/s41416-026-03468-0)
Supplement: Supplementary file 12 — Supplementary Table S5 [file 41416_2026_3468_MOESM12_ESM.docx]

**Supplementary Table S5.** List of antibodies used for immunohistochemical analysis.

| **marker** | **vendor** | **Clone** | **Antigen retrieval** | **Primary antibody incubation** | **dilution** | **Secondary system** |
| --- | --- | --- | --- | --- | --- | --- |
| ER | Roche | SP1 | pH 9, 95^o^C | 36 min | 1:200 | UltraView DAB |
| PR | Novocastra | 16,SAN27 | pH 9, 99^o^C | 32 min | Ready to use | OptiView DAB |
| Ki-67 | Roche | 30-9 | pH 9, 95^o^C | 36 min | Ready to use | UltraView DAB |
| HER-2 | Roche | 4B5 | CE-IVD | | | |
| CK-8 | Biogenex | C51 | pH 9, 98^o^C | 36 min | 1:800 | UltraView DAB |
| E-cadherin | DAKO | NCH-38 | pH 9, 97^o^C | 20 min | 1:200 | EnVision FLEX |
| Vimentin | DCS | SP 20 | pH 9, 97^o^C | 20 min | 1:500 | EnVision FLEX |
| Trop2 | Santa Cruz Biotechnology | B-9 | pH9, 97^o^C | 20 min | 1:50 | EnVision FLEX |
