# Supplementary material for: Circulating tumour cell-derived xenograft as a preclinical platform for metastatic breast cancer
Source: Br J Cancer. 2026 May 18;135(4):568–80. doi: 10.1038/s41416-026-03468-0 (PMC13427727; doi:10.1038/s41416-026-03468-0)
Supplement: Supplementary file 13 — Supplementary Figure legends [file 41416_2026_3468_MOESM13_ESM.docx]

**Kahounová et. al: Circulating tumour cell-derived xenograft as a preclinical platform for metastatic breast cancer**

**DOI:** 10.1038/s41416-026-03468-0

**Supplementary Figure Legends**

**Supplementary Figure S1. Timeline of patient diagnosis, treatment, and blood collection for CTCs isolation. A)** The patient was diagnosed in 2011 at the age of 32 with multicentric invasive ductal carcinoma of the right breast. After adjuvant radiotherapy and tamoxifen treatment, the disease relapsed in 2016 with skeletal metastases, followed by several different treatments. Blood for CTCs isolation was collected from this patient in December 2019. Metastases in the central nervous system appeared shortly after blood collection, and the patient passed away in May 2020. . B) Monitoring changes in the level of cancer antigen 15-3 (CA 15-3) in the blood of the patient over time. C) Timeline of CDX generation and *in vivo* propagation in five consecutive passages. Created in <https://BioRender.com>.

**Supplementary Figure S2. Established CDX_IBP_01 has metastatic potential.**

A full gating strategy from flow cytometry analysis of the metastatic assay (Figure 1E) is presented. Only viable single cells without debris were taken into analysis. CDX-derived metastatic cells were identified as cells negative for mouse lineage markers CD45, CD31, and Ter-119 and simultaneously positive for human surface marker CD298. Representative analysis of lungs from A) an intact animal, and B) an animal bearing a tumor in the mammary fat pad. C) Quantification of CD298^+^ human cancer cells in the primary tumor and the inspected organs from the metastatic assay. Data are presented as % of CD298^+^ events from viable events, from 3 independent experiments performed. D) Detection of xenograft-derived CD298^+^ cancer cells in the primary tumor, in the blood, and in the lungs in two representative mice. Detection of CD298^+^ cells in blood was performed either in whole blood (a) or in the buffy coat (b). E) Expression of Trop-2 on CD298^+^ human cancer cells in primary tumor, blood, and lungs detected by flow cytometry. Results from one representative mouse are shown. F) Immunohistochemical analysis of human Trop-2 expression in the primary tumor in the mammary fat pad and in the lungs. Representative results from the same mouse as in D) are presented. Scale bar 50 µm. The enlarged cut-out is below the corresponding image.

**Supplementary Figure S3. Analysis of selected surface epithelial and mesenchymal markers using spectral flow cytometry.** Data are presented as mean percentage % of positive cells ± SEM for each marker from 3 independent repetitions. One-way ANOVA was used for statistical analysis. * indicates statistical significance p≤0.05, ** indicates statistical significance p≤0.01.

**Supplementary Figure S4. CDX-derived cancer cells share a comparable phenotype *in vivo* and in *in vitro* cell cultures.**

A) t-SNE maps colored by expression profiles of surface antigens from Figure 2 E, F. B) + C) Cluster analysis of data from A) using FlowSOM. 8 populations were identified in the clustered sample from one representative repetition of 2D and 3D *in vitro* cell culture and *in vivo* xenograft. D) Analysis of data from A) using Cluster Explorer. E) Frequency of each population identified in C in 2D and 3D cell culture and in *in vivo* xenograft sample.

**Supplementary Figure S5. Gene expression signatures differentiate the CTC-derived xenograft from the primary tumor.** A) String pathways associating Gene Ontology Biological Processes enriched (left) or lost (right) within CDX_IBP_01 relative to BCa1. B) Dot plot representing Gene Ontology Molecular Functions and C) Cellular Components enriched (left) or lost (right) within CDX_IBP_01 relative to BCa1. The dot size represents the number of genes of a particular biological process; the color intensity represents the fold enrichment (CDX_IBP_01 vs BCa1), and the x-axis determines the -log10 false discovery rate (FDR).

**Supplementary Figure S6.** **CDX reprograms metabolism toward OXPHOS**. A) A plot representing the location of differentially expressed genes on the genome with a zoom into ChrMT and genes changed deregulated between CDX_IBP_01 and BCa1 (right). Red and blue dots represent significantly up-and down-regulated genes, respectively. The distance of the dots from the closest chromosome is proportional to the log2 fold-change (FC). B) Heatmap using expression of representative nuclear- and mitochondrial-encoded genes differentially expressed in CDX_IBP_01 relative to BCa1. C) MitoCarta3.0 and D) KEGG Oxidative Phosphorylation GSEA plots in CDX_IBP_01 vs BCa1 RNA-seq data. E) GSEA plots of KEGG pathways enriched in BCa1 relative to CDX_IBP_01.

**Supplementary Figure S7. CTCs-derived xenograft and *in vitro* culture as tools for drug testing.** A) Schematic workflow of spheroids generation, drug treatment, and image analysis. Created in https://BioRender.com. B) Quantification of the average spheroid area in response to treatment. Data are presented as mean fold of control (vehicle treatment) ± SEM from two independent repetitions performed in technical multiplicates. Arrows indicate days of addition of drugs. C) Quantification of spheroid size at the endpoint (day 21). Data are presented as mean ± SEM from two independent repetitions performed in technical multiplicates. Values for each concentration were normalized to values of the control at day 21. D) Normalized tumor volume of each mouse from 2 independent repetitions. Normalization was performed on the tumor volume at the start of the treatment (day 0). E) Normalized tumor volume presented as mean ± SEM from two independent experiments. Statistical analysis was performed with the Mann-Whitney nonparametric test. F) Quantification of disseminated tumor cells in the lungs of mice at the endpoint (day 21) of the drug treatment experiment. Data are presented as mean ± SEM from one repetition, n=5 for control and carboplatin, and n=4 for vandetanib treatment. Statistical analysis was performed using the Mann-Whitney nonparametric test. The percentage of CD298^+^ cells was calculated from viable cells. See Supplementary Table S4 for the quantification of CD298^+^ cells.
